# Neuropeptide Modulation Enables Biphasic Internetwork Coordination via a Dual-Network Neuron

**DOI:** 10.1523/ENEURO.0121-24.2024

**Published:** 2024-06-18

**Authors:** Barathan Gnanabharathi, Savanna-Rae H. Fahoum, Dawn M. Blitz

**Affiliations:** Department of Biology, Center for Neuroscience and Behavior, Miami University, Oxford, Ohio 45056

**Keywords:** central pattern generator, internetwork, neuromodulation, neuropeptide, rectification, rhythmic

## Abstract

Linked rhythmic behaviors, such as respiration/locomotion or swallowing/chewing, often require coordination for proper function. Despite its prevalence, the cellular mechanisms controlling coordination of the underlying neural networks remain undetermined in most systems. We use the stomatogastric nervous system of the crab *Cancer borealis* to investigate mechanisms of internetwork coordination, due to its small, well-characterized feeding-related networks (gastric mill [chewing, ∼0.1 Hz]; pyloric [filtering food, ∼1 Hz]). Here, we investigate coordination between these networks during the Gly^1^-SIFamide neuropeptide modulatory state. Gly^1^-SIFamide activates a unique triphasic gastric mill rhythm in which the typically pyloric-only LPG neuron generates dual pyloric-plus gastric mill-timed oscillations. Additionally, the pyloric rhythm exhibits shorter cycles during gastric mill rhythm-timed LPG bursts, and longer cycles during IC, or IC plus LG gastric mill neuron bursts. Photoinactivation revealed that LPG is necessary to shorten pyloric cycle period, likely through its rectified electrical coupling to pyloric pacemaker neurons. Hyperpolarizing current injections demonstrated that although LG bursting enables IC bursts, only gastric mill rhythm bursts in IC are necessary to prolong the pyloric cycle period. Surprisingly, LPG photoinactivation also eliminated prolonged pyloric cycles, without changing IC firing frequency or gastric mill burst duration, suggesting that pyloric cycles are prolonged via IC synaptic inhibition of LPG, which indirectly slows the pyloric pacemakers via electrical coupling. Thus, the same dual-network neuron directly conveys excitation from its endogenous bursting and indirectly funnels synaptic inhibition to enable one network to alternately decrease and increase the cycle period of a related network.

## Graphical abstract

**Figure:**
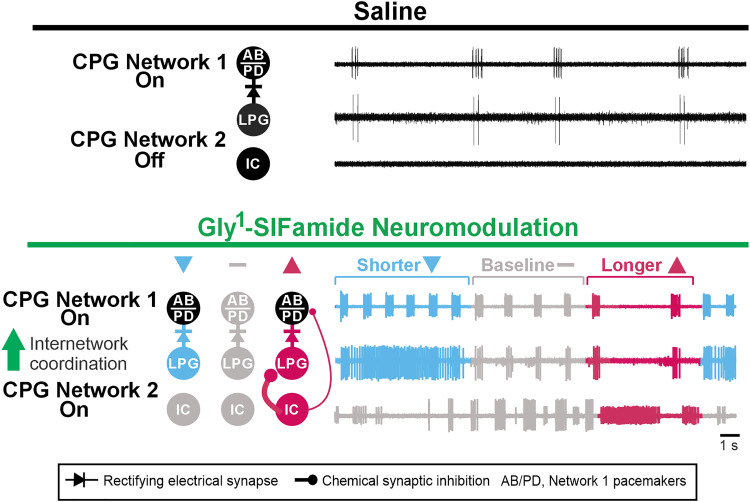
Visual Overview

## Significance Statement

Related rhythmic behaviors frequently exhibit coordination, yet the cellular mechanisms coordinating the underlying neural networks are not determined in most systems. We investigated coordination between two small, well-characterized crustacean feeding-associated networks during a neuropeptide-elicited modulatory state. We find that a dual fast/slow network neuron directly shortens fast network cycles during its slow, intrinsically generated bursts, likely via electrical coupling to fast network pacemakers, despite rectification favoring the opposite direction. Additionally, the fast network is indirectly prolonged during another slow-network phase, via chemical synaptic inhibition that is likely funneled through the same electrical synapse. Thus, a dual-network neuron alternately reinforces and diminishes neuropeptide actions, enabling distinct frequencies of a faster network across different phases of a related slower rhythm.

## Introduction

Many rhythmic behaviors require coordination for proper function, such as locomotion and respiration, and multiple orofacial behaviors (e.g., respiration, swallowing, chewing, vocalization; Cao et al., 2012; Barnett et al., 2021; Hao et al., 2021; Huff et al., 2022; Juvin et al., 2022; Wei et al., 2022). Central pattern generator (CPG) networks controlling rhythmic behaviors are flexible to accommodate changing organismal needs (Bucher et al., 2015; Ramirez and Baertsch, 2018; Grillner and El Manira, 2020). Behavioral coordination requires coordinating the underlying CPG networks. Significant consequences occur from disrupted coordination, such as aspiration of food if swallowing and respiration are not coordinated (Yagi et al., 2017). Thus, it is important to understand the cellular-level mechanisms controlling internetwork coordination.

Network coordination can occur as correlated changes in frequency or strength, as for respiration with changing locomotor speeds or at the onset of vocalizations, or as specific timing relationships to coordinate movements of different body parts (Mulloney and Smarandache-Wellmann, 2012; Daley et al., 2013; Anderson et al., 2016; Ramirez and Baertsch, 2018; Demartsev et al., 2022; Juvin et al., 2022; Wei et al., 2022). Such interactions may occur via direct CPG to CPG projections or indirectly via feedback from networks to higher-order inputs to a related CPG (Bartos and Nusbaum, 1997; Wood et al., 2004; Gariépy et al., 2010). Additional anatomical substrates for network coordination include sensory feedback from one behavior influencing related CPGs or parallel descending input to related networks (Gariépy et al., 2010; Barnett et al., 2021; Juvin et al., 2022). More complex behavioral interactions may also occur, such as variations in respiration during crying, singing, or while playing wind instruments (Fortune et al., 2011; Okobi et al., 2019; Higashino et al., 2022; Wei et al., 2022; Hérent et al., 2023). Further, coordination can be altered by developmental, physiological, or environmental stimuli (Clemens et al., 1998a; Saunders et al., 2004; Blitz and Nusbaum, 2008; Moore et al., 2013; Stein and Harzsch, 2021; Sillar et al., 2023). Although insights have been gained into internetwork coordination, its complexity and plasticity coupled with often large, distributed networks make it difficult to fully determine how network coordination is controlled (Juvin et al., 2022).

**Figure 1.**
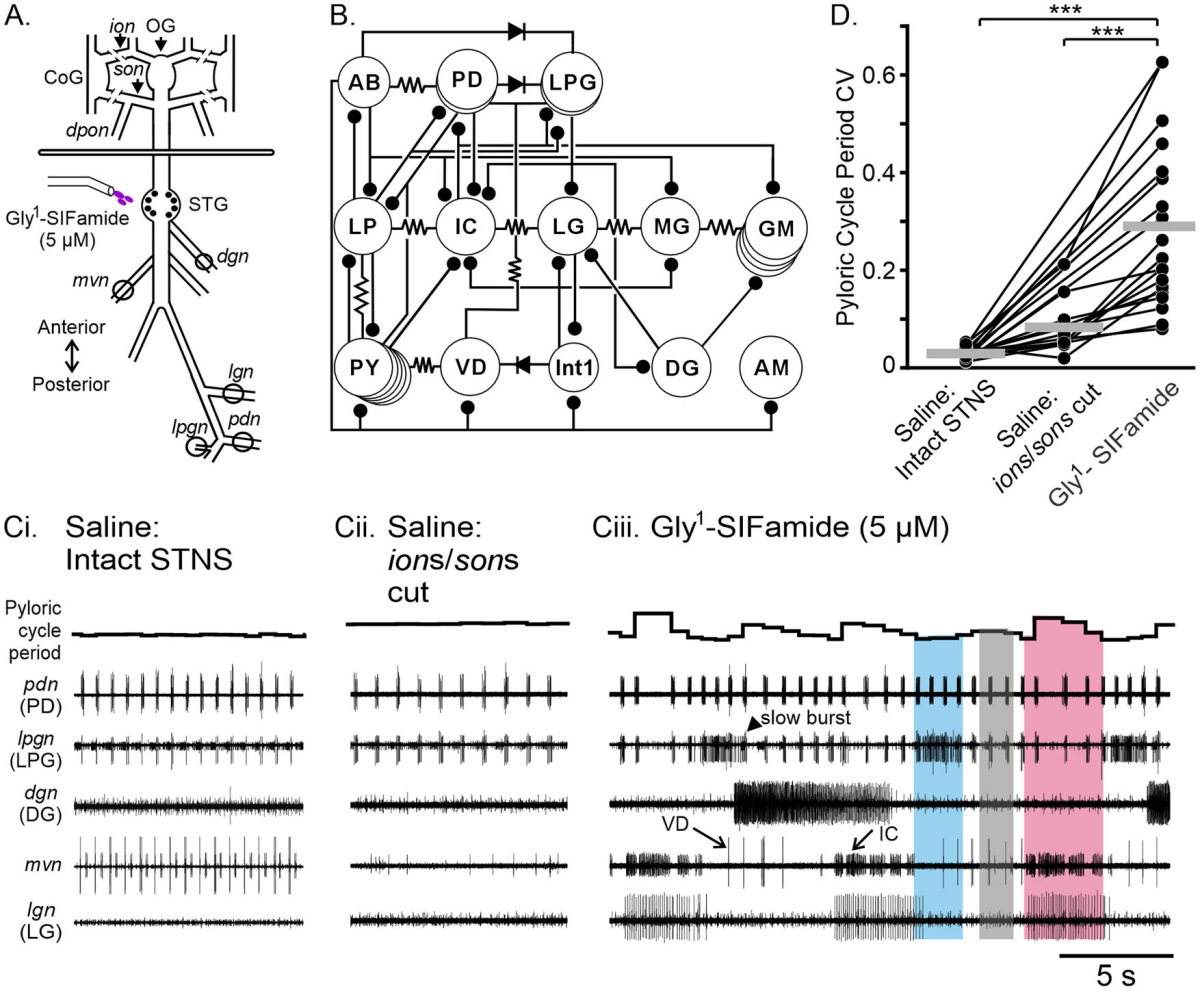
Gly^1^-SIFamide elicited a variable pyloric rhythm. ***A***, Schematic of the isolated stomatogastric nervous system (STNS) of the crab, *Cancer borealis*. The STNS consists of the paired commissural ganglia (CoG), the single oesophageal ganglion (OG), and the single STG, plus motor and connecting nerves. The STG consists of neuropil and the cell bodies of both the gastric mill and pyloric network neurons. The line breaks on the *ion* and *son* indicate where they were cut to isolate the STG network (Saline:*ions/sons* cut). Two compartments were created with a Vaseline wall across the dish and separately superfused. Gly^1^-SIFamide (5 µM) was bath applied selectively to the posterior compartment containing the STG. ***B***, Schematic of the pyloric and gastric mill connectome. Resistor symbols indicate electrical coupling, diode symbols indicate rectification of electrical coupling, and ball and stick symbols indicate chemical inhibition. ***C***, Representative electrophysiological traces of the pyloric (*pdn*, *mvn*, *lpgn*) and gastric mill (*mvn*, *dgn*, and *lgn*) networks in an example experiment in Saline:Intact STNS (***Ci***), Saline:*ions/sons* cut (***Cii***), and bath-applied Gly^1^-SIFamide (5 µM; ***Ciii***) conditions. The instantaneous pyloric cycle period is plotted at the top of each set of traces. All conditions are from the same experiment. The colored boxes indicate three levels of pyloric cycle period, overlapping with three distinct phases of the Gly^1^-SIFamide gastric mill rhythm (blue, LPG activity; pink, IC/LG activity; gray, baseline Gly^1^-SIFamide modulation when no gastric mill neurons are active; see also Fig. 2). ***D***, The CV of the pyloric cycle period is plotted for three experimental conditions: (1) in saline prior to isolation of the STG network (Saline:Intact STNS, *n* = 19), (2) in saline with *ion*s*/son*s cut to isolate the STG network from descending modulatory inputs (Saline:*ion*s/*son*s cut, *n* = 12) and (3) during 5 µM Gly^1^-SIFamide bath application (SIFamide, *n* = 19). Each dot represents individual experiments and lines connecting across the experimental conditions depict data points from the same preparation. The different *n*-value for *ion*s/*son*s cut is due to the lack of a pyloric rhythm in seven preparations in this condition. In the absence of modulatory inputs, the pyloric rhythm sometimes shuts off (Zhang et al., 2009; Hamood et al., 2015). ***p < 0.001, one-way RM ANOVA, Holm–Sidak *post hoc*. Neurons: AB, anterior burster; AM, anterior median; DG, dorsal gastric; GM, gastric mill; IC, inferior cardiac; Int1, Interneuron 1; LG, lateral gastric; LP, lateral pyloric; LPG, lateral posterior gastric; MG, medial gastric; PD, pyloric dilator; PY, pyloric; VD, ventricular dilator. Nerves: *dgn*, dorsal gastric nerve; *ion*, inferior oesophageal nerve; *lgn*, lateral gastric nerve; *lpgn*, lateral posterior gastric nerve; *lvn*, lateral ventricular nerve; *mvn*, median ventricular nerve; *pdn*, pyloric dilator nerve; *son*, superior oesophageal nerve; *stn*, stomatogastric nerve.

Here we used small, well-described networks in the stomatogastric nervous system (STNS) of the Jonah crab, *Cancer borealis*, to investigate cellular-level mechanisms of internetwork coordination (Bartos and Nusbaum, 1997; Bartos et al., 1999; Wood et al., 2004; Daur et al., 2016). An in vitro STNS preparation includes the stomatogastric ganglion (STG, ∼30 neurons) containing well-characterized CPGs that control the gastric mill (food chewing) and pyloric (food filtering) rhythms, plus identified modulatory and sensory inputs (Fig. 1; Nusbaum and Beenhakker, 2002; Daur et al., 2016; Blitz, 2023). The pacemaker-driven pyloric rhythm is constitutively active in vivo and in vitro (∼1 s cycle period), whereas the slower (∼10 s cycle period) network-driven gastric mill CPG requires activation by modulatory or sensory inputs (Nusbaum and Beenhakker, 2002; Blitz, 2023). All pyloric and gastric mill neurons (1–5 neurons/type) and their transmitters are identified, and their connectivity is mapped under multiple modulatory conditions (Fig. 1*B*; Marder and Bucher, 2007; Daur et al., 2016; Fahoum and Blitz, 2024). Although the gastric mill and pyloric rhythms can occur independently, they are often coordinated, and the extent and type of coordination is altered by modulatory, behavioral, and environmental conditions (Bartos and Nusbaum, 1997; Clemens et al., 1998a; Bartos et al., 1999; Wood et al., 2004; Blitz et al., 2019; Stein and Harzsch, 2021).

We investigated mechanisms of coordination during the modulatory state elicited by the modulatory commissural neuron 5 (MCN5) or bath application of its neuropeptide Gly^1^-SIFamide (Blitz et al., 2019; Fahoum and Blitz, 2021, 2024). In this state, the typically pyloric-only LPG switches into dual pyloric and gastric mill rhythm participation, becoming the third phase of a gastric mill rhythm (Fahoum and Blitz, 2024). This contrasts with biphasic gastric mill rhythm versions (Beenhakker et al., 2004; Christie et al., 2004; Blitz et al., 2008, 1999). The pyloric rhythm cycle period varies across the Gly^1^-SIFamide gastric mill rhythm, but a full description plus identification of mechanisms of interactions between the two networks is lacking (Blitz et al., 2019). The well-described connectome and small populations enabled selective manipulation of neuronal populations to determine the full extent of gastric mill regulation of the pyloric rhythm and its underlying cellular mechanisms during the unique triphasic Gly^1^-SIFamide-elicited gastric mill rhythm. Our results highlight the complexity of synaptic connectivity that can mediate coordination between related networks.

## Materials and Methods

### Animals

Wild caught adult male *C. borealis* crabs were procured from The Fresh Lobster Company, maintained in artificial seawater (10–12°C) tanks, and fed twice weekly until used. Prior to dissection, crabs were anesthetized by cold packing in ice for 40–50 min. The crab foregut was first removed, bisected, and pinned flat, ventral side up, in a Sylgard 170-lined dish (Thermo Fisher Scientific). The STNS was then dissected free from muscles and connective tissue and pinned in a Sylgard 184-lined petri dish (Thermo Fisher Scientific; Gutierrez and Grashow, 2009; Fahoum and Blitz, 2021). The preparation was kept in chilled (4°C) *C. borealis* physiological saline throughout dissections.

### Solutions

*C. borealis* physiological saline was composed of the following (mM): 440 NaCl, 26 MgCl_2_, 13 CaCl_2_, 11 KCl, 10 Trizma base, 5 maleic acid, pH 7.4–7.6. Squid internal electrode solution contained the following (in mM): 10 MgCl_2_, 400 potassium d-gluconic acid, 10 HEPES, 15 Na_2_SO_4_, 20 NaCl, pH 7.45 (Hooper et al., 2015). Gly^1^-SIFamide (GYRKPPFNG-SIFamide, custom peptide synthesis: GenScript; Huybrechts et al., 2003; Yasuda et al., 2004; Dickinson et al., 2008; Blitz et al., 2019) was prepared by dissolving it in optima water (Thermo Fisher Scientific) at 10 mM and aliquoting and storing it at −20°C. Aliquots were diluted in physiological saline to a final concentration of 5 µM before use in experiments.

### Electrophysiology

The STNS preparation was superfused continuously with chilled *C. borealis* physiological saline (8–10°C) or Gly^1^-SIFamide (5 µM) diluted in saline. A switching manifold enabled uninterrupted superfusion of the STNS preparation during solution changes. Extracellular activity was recorded from nerves with custom stainless-steel pin electrodes and a model 1700 A-M Systems Amplifier. The STG was desheathed to gain access for intracellular recording of STG somata. Light transmitted through a dark-field condenser (MBL-12010 Nikon Instruments) provided visualization of STG neuron somata. For intracellular recordings of STG neurons, sharp-tip glass microelectrodes pulled using a glass electrode puller (P-97, Flaming/Brown microelectrode puller, Sutter Instrument) were used. Microelectrodes were filled with a squid internal solution (see above, Solutions; resistance, 20–40 MΩ; Hooper et al., 2015). Axoclamp 900A amplifiers (Molecular Devices) were used to collect intracellular recordings in current-clamp mode. Recordings were collected before, during, and after the transection of both inferior and superior oesophageal nerves (*ion* and *son,* respectively) to isolate the STG from descending neuromodulatory inputs. Data presented were collected after transections were performed, unless otherwise noted. For intracellular recordings, STG neuronal cell bodies were identified using extracellular nerve recordings and/or their interactions with other STG neurons. All recordings were collected using acquisition hardware (Micro1401; Cambridge Electronic Design) and software (Spike2 version 8.02e; ∼5 kHz sampling rate; Cambridge Electronic Design) and a laboratory computer (Dell).

A dataset from Fahoum and Blitz (2021) was reused in this study for different measurements than the previous study. As described, activity in the inferior cardiac (IC), lateral gastric (LG), or dorsal gastric (DG) neurons, or all three neurons simultaneously, was eliminated via hyperpolarizing current injection (−2 to −4 nA). Neurons were hyperpolarized sufficiently to eliminate spike-mediated and graded transmitter release (Fahoum and Blitz, 2021).

For the established biological model of MCN5 actions, it is necessary to eliminate LP activity to mimic the MCN1 glutamatergic inhibition of LP (Fahoum and Blitz, 2021). Therefore, for all experiments using bath application of Gly^1^-SIFamide (5 µM), the lateral pyloric (LP) neuron was either hyperpolarized (−2 to −4 nA) or photoinactivated (Fahoum and Blitz, 2021; Snyder and Blitz, 2022). To selectively eliminate the influence of the single LP neuron or the two copies of the LPG neurons, neurons were impaled with a microelectrode that was tip-filled with Alexa Fluor 568 hydrazide (10 mM in 200 mM KCl; Thermo Fisher Scientific) and backfilled with squid internal solution. The neurons were injected with −5 nA hyperpolarizing current (for either ∼ 30 min or for 5–10 min and the dye then allowed to diffuse for an additional ∼30 min) to fill their soma and neurites with the Alexa Fluor 568 dye. The STG was then illuminated with a Texas red filter set (560 ± 40 nm wavelength; Leica Microsystems, 3–7 min). The neurons were considered completely photoinactivated when the membrane potential reached 0 mV, and their action potentials were absent in either the lateral ventricular nerve (*lvn;* for LP) or the lateral posterior gastric nerve (*lpgn;* for LPG).

### Data analysis

#### Neuronal activity quantification

The number of bursts, burst duration (s), cycle period (s; start of one burst to the start of the subsequent burst of a reference neuron), number of spikes per burst, and firing frequency (Hz; [number of spikes per burst − 1] / burst duration) were quantified using a custom Spike2 script. Each activity parameter was averaged across 20 min, unless otherwise noted. All analyses were performed after the effects of Gly^1^-SIFamide application reached steady state. Steady state was noted when LPG elicited consistent dual-network bursting (Fig. 1*Ciii*) with consistent gastric mill-timed bursting of LG, IC, and DG (∼ 10 min from the start of Gly^1^-SIFamide bath application). The pyloric cycle period was quantified as the duration (s) from the start of one pyloric dilator (PD) neuron burst to the start of the subsequent PD burst (Bartos et al., 1999). In the Saline:*ions*/*sons* cut condition, in which the *ions/sons* were transected, the pyloric cycle period did not always express a full triphasic pattern, due to the lack of modulatory input (Zhang et al., 2009; Hamood et al., 2015; Spencer and Blitz, 2016). However, when this occurred, the pyloric rhythm returned during the Gly^1^-SIFamide bath application. A Saline:*ions/sons* cut condition was excluded from analysis if PD neuron bursting was intermittently active, was completely absent, or if the PD neurons were rhythmically active but had ≤2 action potentials per burst. Pyloric cycle periods were measured across the final 20 min of 30 min Gly^1^-SIFamide (5 µM) bath applications and across 200 s windows for Saline:Intact STNS and the Saline:*ions/sons* cut conditions.

Gastric mill-timed IC bursts were identified as those that had burst durations >0.45 s (Blitz et al., 2019; Fahoum and Blitz, 2024). Multiple IC bursts with >0.45 s burst durations were grouped into a single “gastric mill” burst if the distance between each burst was <2 s; otherwise they were considered as individual bursts. If there was an IC burst with a burst duration <0.45 s between two gastric mill IC bursts that were <2 s apart, they were grouped together as one gastric mill burst. Within an IC gastric mill-timed burst, pyloric-timed IC bursts were identified if there was an interruption timed to a PD burst (Fig. 6*Ai*–*ii*). We identified LPG gastric mill-timed bursts (LPG slow bursts) as previously described (Fahoum and Blitz, 2021, 2024; Snyder and Blitz, 2022). Briefly, from a histogram of all LPG interspike intervals (ISIs) made in Excel (Microsoft), peaks of intraburst (interval between spikes during a burst) and interburst (interval between spikes between burst) intervals were identified. The mean ISI was calculated between these two peaks and used to identify LPG bursts. A custom-written Spike2 script was then used to identify LPG gastric mill-timed slow bursts and exclude pyloric-timed LPG bursts using PD activity as a reference (Fahoum and Blitz, 2021). Coefficient of variation (CV) of pyloric cycle periods was calculated as standard deviation/mean.

#### Analysis of pyloric cycle period relative to gastric mill neuron activity

To analyze pyloric cycle periods based on which gastric mill neurons were active, burst timings for each neuron over a 20 min window, unless otherwise indicated, were exported from Spike2 to MATLAB (MathWorks) for further analysis. A custom-written MATLAB script categorized PD cycle periods according to which gastric mill neuron(s) bursts overlapped with each pyloric cycle. This included any amount of overlap between a pyloric cycle period and a gastric mill neuron burst. Pyloric cycle periods that did not overlap with any gastric mill neurons were placed into the SIFbaseline category, i.e., no gastric mill neurons active. Pyloric cycle periods assigned to a particular category were averaged for each preparation, and a total average was then calculated across all experiments for each category. In some preparations, no pyloric cycle periods were assigned to some of the categories. Specifically, for IC, LPG:IC, and LPG:DG:IC, there were 9–10 preparations that had no cycle periods assigned to each of these categories while for the remaining 13 categories there were 0–3 preparations that had no pyloric cycles assigned to these categories. This dataset (*n* = 16/19 from Fahoum and Blitz, 2021) and the LPG:Intact and LPG:Kill condition experiments (*n* = 9/9 from Fahoum and Blitz, 2024) were reanalyzed from previous studies. Two experiments from the original dataset of 11 were excluded from analysis due to a lack of biphasic regulation in the LPG:Intact condition.

Due to the interpreparation variability of the pyloric cycle period, to test for changes in regulation of the pyloric cycle period between LPG:Kill and LPG:Intact conditions, all pyloric cycle periods were normalized to the average pyloric cycle period in the SIFbaseline category in each experiment. All pyloric cycle periods after normalization were plotted as a histogram with a bin size of 3 ms for both conditions. Further, bin counts for each histogram were normalized to the peak count in that condition to compare the overall distribution of the two histograms. Normalization and histogram plotting were performed using a custom-written MATLAB script.

### Figure preparation and statistical analysis

Burst and spike identification from raw data was performed with custom-written scripts in Spike2 and exported to either Microsoft Excel or MATLAB for further analysis. Pyloric cycle period histograms were plotted using a script written in MATLAB. Figures were created using graphs and recordings exported from MATLAB, SigmaPlot (Systat, v13), and Spike2 into CorelDRAW (Corel, v24). Statistical analyses were performed using SigmaPlot software and R statistical software (v4.1.1). Data were analyzed for normality using the Shapiro–Wilk test to determine whether a nonparametric or parametric test would be used. Paired *t* test, one-way repeated measures ANOVA, mixed model ANOVA, and *post hoc* tests were used as noted. For all tests, we used an alpha level of 0.05. Data are presented as mean ± SE.

### Code accessibility

Spike2 and MATLAB scripts used for analysis are available at https://github.com/blitzdm/GnanabharathiFahoumBlitz2024.

## Results

### Gly^1^-SIFamide elicits a variable pyloric rhythm

The modulatory projection neuron MCN5 elicits a unique gastric mill rhythm that can be reproduced in vitro by bath application of the MCN5 neuropeptide Gly^1^-SIFamide plus elimination of LP neuron activity (Blitz et al., 2019; Fahoum and Blitz, 2021, 2024). MCN5 inhibits LP via its cotransmitter glutamate, but Gly^1^-SIFamide bath application excites LP, likely mimicking the actions of a second Gly^1^-SIFamide-containing input to the STG (Blitz et al., 2019; Fahoum and Blitz, 2021). Therefore, we hyperpolarized or photoinactivated the LP neuron to enable LPG dual-network activity and better mimic the MCN5-elicited motor pattern. In *C. borealis*, LPG is typically only active with the pyloric network due to its electrical coupling with the rest of the pyloric pacemaker ensemble (Fig. 1*B*,*C*; Shruti et al., 2014) but switches into dual-network activity characterized by longer duration, slower gastric mill-timed bursts alternating with shorter duration, faster pyloric-timed bursts during Gly^1^-SIFamide application (Fig. 1*Ciii*; Blitz et al., 2019; Fahoum and Blitz, 2021, 2024). The unique Gly^1^-SIFamide gastric mill rhythm is characterized by coactivity of LG and IC neurons that are primarily out of phase with DG neuron activity, followed by LPG neuron gastric mill activity (Blitz et al., 2019; Fahoum and Blitz, 2024; Fig. 1*C*). Anecdotally, the pyloric rhythm cycle period varies throughout the Gly^1^-SIFamide gastric mill rhythm, with a correlation between IC burst duration and extended duration pyloric cycles (Blitz et al., 2019; Fahoum and Blitz, 2021). Here we investigate in detail the timing relationship between pyloric and gastric mill rhythms in the Gly^1^-SIFamide modulatory state.

We first quantified the variability of the pyloric rhythm by measuring the CV in control and during the Gly^1^-SIFamide gastric mill rhythm. To eliminate confounds due to a potentially variable set of endogenously active modulatory inputs, we transected the *ion*s and *son*s to obtain the same baseline condition across preparations. However, this often results in a very slow pyloric rhythm or the pyloric rhythm shutting off entirely (Zhang et al., 2009; Hamood et al., 2015). Therefore, to compare the variability of the pyloric rhythm during Gly^1^-SIFamide to a similarly robust pyloric rhythm, we included a comparison of the pyloric rhythm in saline in the intact STNS, in addition to during saline superfusion after *ion*/*son* transection. In an example experiment, the pyloric cycle period was ∼1.6 s, with a CV of 0.02 (Fig. 1*Ci*, PD activity in the *pdn*, instantaneous cycle period plotted above traces). After *ion*/*son* transection, the pyloric cycle period increased to ∼ 2.4 s and a CV of 0.05 (Fig. 1*Cii*). During Gly^1^-SIFamide, a gastric mill rhythm was activated, evident in LG and DG neuron bursting (Fig. 1*Ciii*, *lgn*, *dgn* recordings) and longer duration gastric mill-timed bursting in the IC neuron (*mvn*). Additionally, the LPG neuron switched from pyloric-only activity (Fig. 1*Ci–ii*; *lpgn*, saline conditions) to dual pyloric and gastric mill (slow) bursting (Fig. 1*Ciii*). Although the average pyloric cycle period in Gly^1^-SIFamide (1.6 s) was similar to the intact STNS in this example, there was a visually evident increase in variability (CV = 0.22; Fig. 1*Ciii*). The increased variability appeared to be due to three levels of pyloric cycle period (Fig. 1*Ciii*, colored boxes, discussed in more detail below). When quantified across preparations, we found a consistent increase in pyloric cycle period variability in Gly^1^-SIFamide compared with saline, including both Saline:Intact STNS and after Saline:*ions*/*sons* cut (Fig. 1*D*; one-way RM ANOVA; *F*_(18,2)_ = 30.09; *p* < 0.001; Table 1). In some experiments, there was no active pyloric rhythm to quantify in the Saline:*ions/sons* cut condition, and thus points between the Saline:Intact STNS and Gly^1^-SIFamide conditions are connected without an intermediate (Saline:*ion*s/*son*s cut) data point (see Materials and Methods). Thus, compared with the pyloric rhythm when influenced by endogenously active modulatory inputs (Saline:Intact STNS), or a slower pyloric rhythm in the absence of modulatory inputs, the pyloric rhythm cycle period was more variable in the Gly^1^-SIFamide modulatory state.

**Table 1.** Pyloric cycle period CV in saline (intact STNS or with *ion*s/*son*s transected) and during bath application of Gly^1^-SIFamide (5 μM)

Pyloric cycle period CV mean ± SEM				*p* value		
Saline:Intact STNS (*n* = 19)	Saline:*ion*/*son* cut (*n* = 12)	Gly^1^-SIFamide (*n* = 19)	*F* (df)	Saline:Intact STNS vs SIFamide	Saline:*ion*/*son* cut vs SIFamide	Saline:Intact STNS vs Saline:*ion*/*son* cut
0.03 ± 0.002	0.08 ± 0.01	0.29 ± 0.04	30.09 (18,2)	<0.001	<0.001	0.182

One-way repeated measures ANOVA, Holm–Sidak *post hoc*; CV, coefficient of variation; df, degrees of freedom.

### Biphasic regulation of the pyloric rhythm

The larger pyloric cycle period CV during Gly^1^-SIFamide application appeared to be due to rhythmic changes in cycle period coinciding with activity in some of the gastric mill neurons. Specifically, in example experiments, the pyloric cycle period was longer during times when the IC and LG neurons were active (Figs. 1*Ciii*, 2*A*, pink box) relative to pyloric activity when no gastric mill neuron was active (Figs. 1*Ciii*, 2*A*; SIFbaseline; gray box; dotted horizontal line across plot of instantaneous cycle period). Shorter duration pyloric cycle periods relative to SIFbaseline coincided with LPG neuron slow bursts (Figs. 1*Ciii*, 2*A*, blue box).

**Figure 2.**
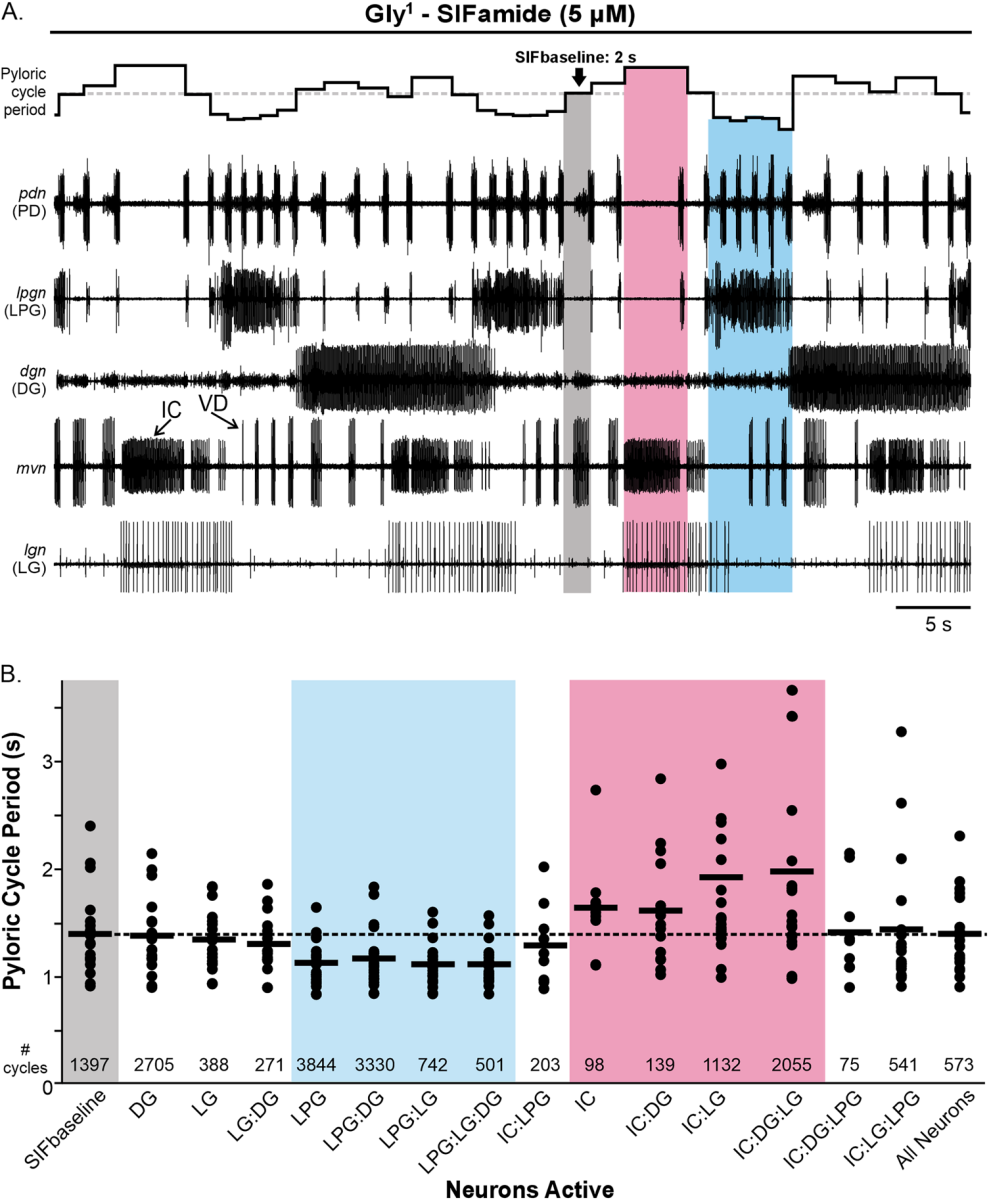
Pyloric cycle periods occurred at three different levels during the Gly^1^-SIFamide gastric mill rhythm. ***A***, The pyloric rhythm occurs with a shorter cycle period during LPG activity (blue box), with a longer cycle period during LG:IC activity (pink box) and an intermediate level in the absence of any gastric mill-timed bursting (SIFbaseline; gray box). Representative traces show an example rhythm during Gly^1^-SIFamide bath application, with instantaneous pyloric cycle period plotted at the top. Dotted line across instantaneous cycle period plot indicates SIFbaseline cycle period. ***B***, The average pyloric cycle period is plotted for no gastric mill neuron activity (SIFbaseline; gray box) and during activity of each combination of 1, 2, 3, and 4 gastric mill neurons (*n* = 19). Each dot represents the average pyloric cycle period during that particular neuron activity across a 20 min analysis window during Gly^1^-SIFamide steady state in a single experiment. Black bars indicate the mean pyloric cycle period across all experiments during that neuronal activity. Numbers at the bottom of the graph depict the total number of pyloric cycles contributing to the averages for that neuronal activity. Dotted line represents average pyloric cycle period during SIFbaseline (gray box). Blue box highlights neuronal activity combinations that include LPG and pink box highlights combinations that include IC.

To further examine the relationship between pyloric cycle period and gastric mill neurons, we measured all pyloric cycle periods across the final 20 min steady state of 30 min Gly^1^-SIFamide (5 µM) bath applications and grouped them by the gastric mill neurons that were active. Considering the four gastric mill neurons (LG, LPG, IC, DG), plus the SIFbaseline condition in which no gastric mill neurons are active, there are 16 possible combinations of active gastric mill neurons during which a pyloric cycle period might have occurred (Fig. 2*B*). We focused only on gastric mill-timed bursts (see Materials and Methods; Fahoum and Blitz 2024), including for IC and LPG which express dual-network activity. The average cycle period occurring during each neuron or combination of neurons active, per experiment (black dots), and across all experiments (black bars) are plotted for the 16 different combinations (Fig. 2*B*). Across 19 preparations, there were some preparations in which no pyloric cycle periods overlapped with a combination of neurons. Specifically in the IC, LPG:IC, and LPG:DG:IC categories, there were between 9 and 10 preparations with no pyloric cycle periods falling into these categories. In the 13 remaining categories, there were 0–3 preparations that had no pyloric cycles in these categories. The total number of pyloric cycles that occurred during each condition, across 19 preparations, are indicated above the *x*-axis labels (Fig. 2*B*, # cycles).

We first visually assessed cycle periods as a function of gastric mill neurons active, using a dashed line to highlight the average cycle period across preparations during the SIFbaseline, when no gastric mill neurons were active (gray region, dashed line). Relative to this baseline, the average cycle period per experiment (dots) tended to be shorter in duration than SIFbaseline during LPG activity, whether it was LPG alone or in combination with DG and/or LG neurons (blue region), including the cumulative average cycle period across preparations (black bars; *n* = 16–19; Fig. 2*B*). Conversely, average cycle periods during combinations of IC, DG, and LG activity (pink region) tended to be longer, and thus above SIFbaseline in the graph, including the cumulative average cycle period (IC, *n* = 9; IC:DG, IC:LG, IC:DG:LG, *n* = 19). This aligns with an earlier study in which longer pyloric cycle periods correlated with longer duration IC bursts (Blitz et al., 2019). Based on the number of cycles, IC gastric mill-timed bursts most often coincided with LG activity (Fig. 2*B*, # cycles). Overall, this representation of cycle period as a function of gastric mill neuron activity suggests that LPG, IC, and LG activity may be responsible for the increased variability in pyloric cycle period during the Gly^1^-SIFamide gastric mill rhythm compared with saline (Fig. 1*D*). To analyze this dataset statistically, we used a mixed model ANOVA to accommodate the large number of conditions, i.e., combinations of gastric mill neurons active, plus missing values for some combinations (*n* = 9–19).

For the statistical analysis of gastric mill neuron activity on the pyloric cycle period, we considered each gastric mill neuron to be a factor with a full four-factor within-subjects mixed model ANOVA, including all higher-order interactions. This model indicated statistically significant effects of gastric mill neuron combinations on pyloric cycle period variability ( $X_{\mathrm{df}=15}^{2}$_ _= 102.57; *p* < 0.0001). The model complexity was then iteratively paired down, eliminating nonsignificant higher-order interactions (Table 2). The most parsimonious model that still explained the variability in pyloric cycle period included single factors LPG, IC, and LG and two-factor terms IC:LPG and IC:LG (Table 2). In this model, there were significant interactions between LG and IC ( $X_{\mathrm{df}=239}^{2}$_ _= 3.07; *p* = 0.002) and between LPG and IC ( $X_{\mathrm{df}=238}^{2}$_ _= −2.38; *p* = 0.02). Specifically, LG had an effect on pyloric cycle period when IC was firing a gastric mill burst (*p* = 0.0011; Table 2) but not without IC gastric mill activity (*p* = 0.40; Table 2). However, IC had an effect on cycle period with (*p* < 0.0001) or without LG activity (*p* = 0.48; Table 2), although a weaker effect in the absence of LG. This points to IC playing an important role in the longer cycle pyloric cycle periods, with possible assistance from LG. For the LPG and IC interaction, LPG had an effect on pyloric cycle period with (*p* < 0.0001) or without (*p* = 0.0004; Table 2) IC generating a gastric mill burst. Similarly, IC affected the pyloric cycle period with (*p* = 0.008) or without (*p* < 0.0001; Table 2) LPG firing a gastric mill burst. Thus, from the overlap in timing and the preceding statistical analysis, it appears that IC (possibly LG) and LPG are responsible for the increased and decreased cycle period, respectively, and the consequent higher cycle period variability during the Gly^1^-SIFamide gastric mill rhythm. We therefore tested the roles of IC, LG, and LPG in regulating pyloric cycle period by manipulating their activity.

### IC, but not LG, is responsible for longer pyloric cycle periods

**Figure 3.**
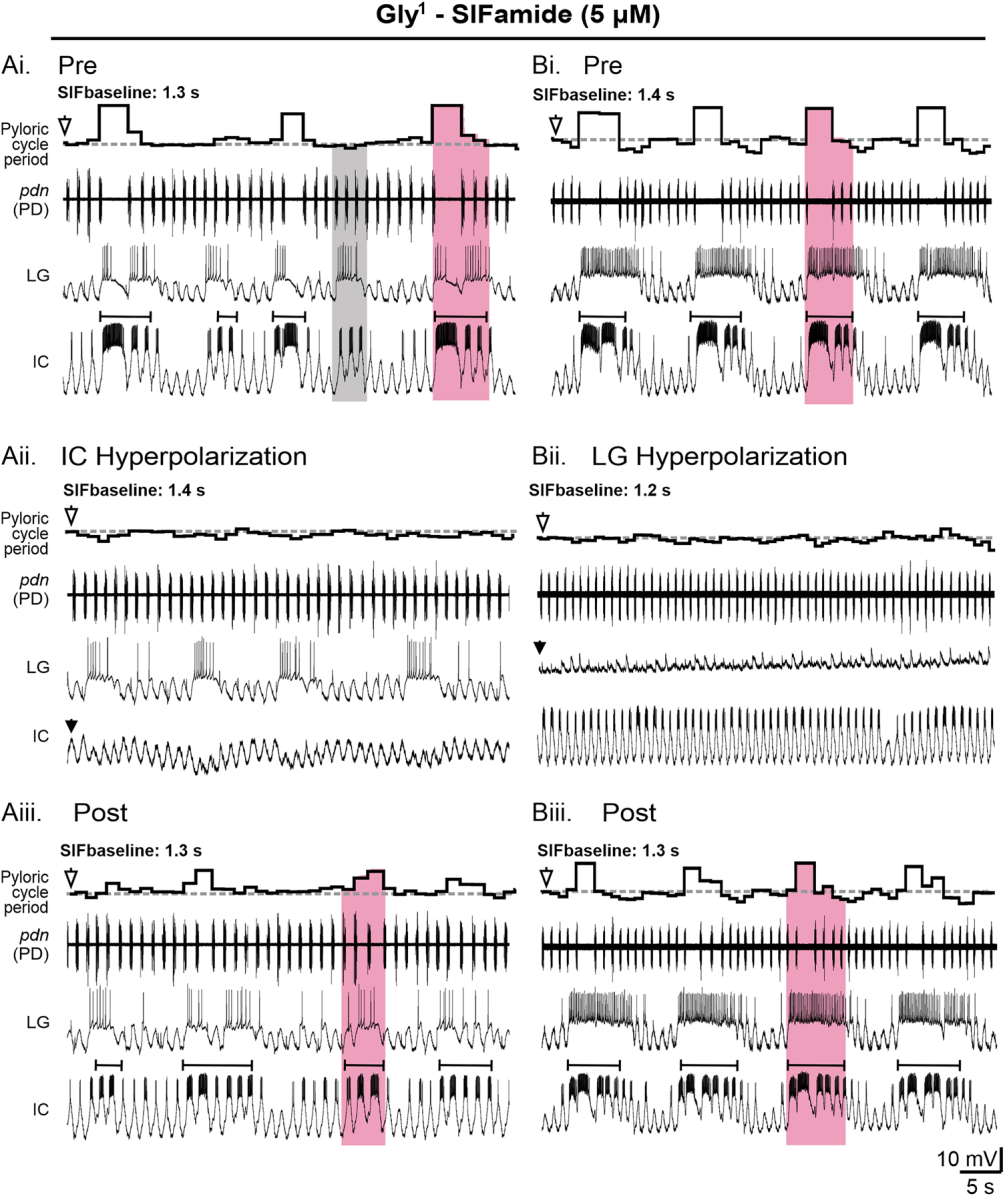
IC or LG hyperpolarization eliminated longer cycle periods and decreased pyloric cycle period variability. ***A***, Extracellular and intracellular recordings monitor the pyloric rhythm (*pdn*) and gastric mill-timed bursting in the IC and LG neurons in control (Pre; ***Ai***, ***Bi***; top), during IC (***Aii***) or LG (***Bii***) hyperpolarization, and after hyperpolarization (Post; ***Aiii***, ***Biii***), all during Gly^1^-SIFamide bath application. ***A*** and ***B*** are from different experiments. Pyloric cycle period on top of the extracellular *pdn* recording plots the instantaneous pyloric cycle period during each condition. Note the absence of longer pyloric cycle periods during both LG and IC hyperpolarization (pink boxes indicate LG:IC activity, gray box indicates LG activity without an IC gastric mill burst (***Ai***). IC gastric mill bursts are identified by brackets above the IC recordings. Downward filled arrowheads indicate hyperpolarizing current injection (***Aii*,*Bii***). Downward white arrowheads above instantaneous pyloric cycle period indicate the SIFbaseline cycle period (dashed line).

We first addressed whether IC and/or LG were necessary for longer pyloric cycle periods during the Gly^1^-SIFamide gastric mill rhythm. The pyloric cycle period was variable in control conditions as is evident in two example experiments (Fig. 3*Ai*,*Bi*, *pdn*, instantaneous cycle period plot above traces). This included longer pyloric cycle periods during gastric mill-timed coactivity in LG and IC (Fig. 3, pink boxes). Identified IC gastric mill bursts (see Materials and Methods; Fahoum and Blitz, 2024) are marked with brackets. In one instance in the time range shown, when LG fired a gastric mill burst, but IC produced only pyloric-timed activity that did not reach the level of a gastric mill-timed burst (Fig. 3*Ai*, gray box), there was no extension of the pyloric cycle period. This is consistent with the statistical analysis of data from Figure 2*B* that LG had an effect on pyloric cycle period, only when IC was also active (in gastric mill time). To test whether IC and/or LG was responsible for the longer pyloric cycle periods, we reanalyzed a dataset from Fahoum and Blitz (2021) in which hyperpolarizing current (−2 to −4 nA) was injected into either IC or LG neurons. In the example experiments, the much longer cycle periods were eliminated when either LG or IC was hyperpolarized (Fig. 3*Aii*,*Bii*). When IC was hyperpolarized, LG bursting persisted and was qualitatively similar to pre-hyperpolarization (Fig. 3*Ai*–*ii*). However, when LG was hyperpolarized, IC did not generate any gastric mill-timed bursts, instead its activity was entirely pyloric timed (Fig. 3*Bii*). Longer pyloric cycle periods, and therefore larger cycle period variability, returned post-hyperpolarization, as did IC gastric mill-timed bursting upon removal of hyperpolarizing current into LG (Fig. 3*Aiii*,*Biii*).

**Table 2.** Statistical analysis of active gastric mill neuron contributions to pyloric cycle period variability during Gly^1^-SIFamide (5 μM) application

Model complexity		X^2(^^a^^,^^b^^)^, df t^c^, df	*p* value
Four-factor interaction term removed		0.0612^a^, 1	0.8047
All 3-factor interaction terms removed		2.9444^b^, 4	0.5672
Final Model:		1.019^b^, 5	0.961
Includes LPG, LG, IC, LPG:IC, LG:IC		4.015^a^, 10	0.946
Post hoc comparisons of significant 2-factor interactions in final model	LG with IC present	−3.30^c^, 240	0.011
	LG with IC absent	0.84^c^, 239	0.40
	IC with LG present	−7.15^c^, 238	<0.0001
	IC with LG absent	−1.99^c^, 241	0.048
	LPG with IC present	−6.35^c^, 238	<0.0001
	LPG with IC absent	−3.57^c^, 238	0.0004
	IC with LPG present	−2.69^c^, 239	0.008
	IC with LPG absent	−6.23^c^, 238	<0.0001

Four-factor within-subjects mixed model ANOVA with iterative elimination of nonsignificant higher-order interactions. Models were compared with full model^a^ or previous level of complexity^b^ using a likelihood ratio test. ^c^*Post hoc* comparisons of estimated marginal means.

**Figure 4.**
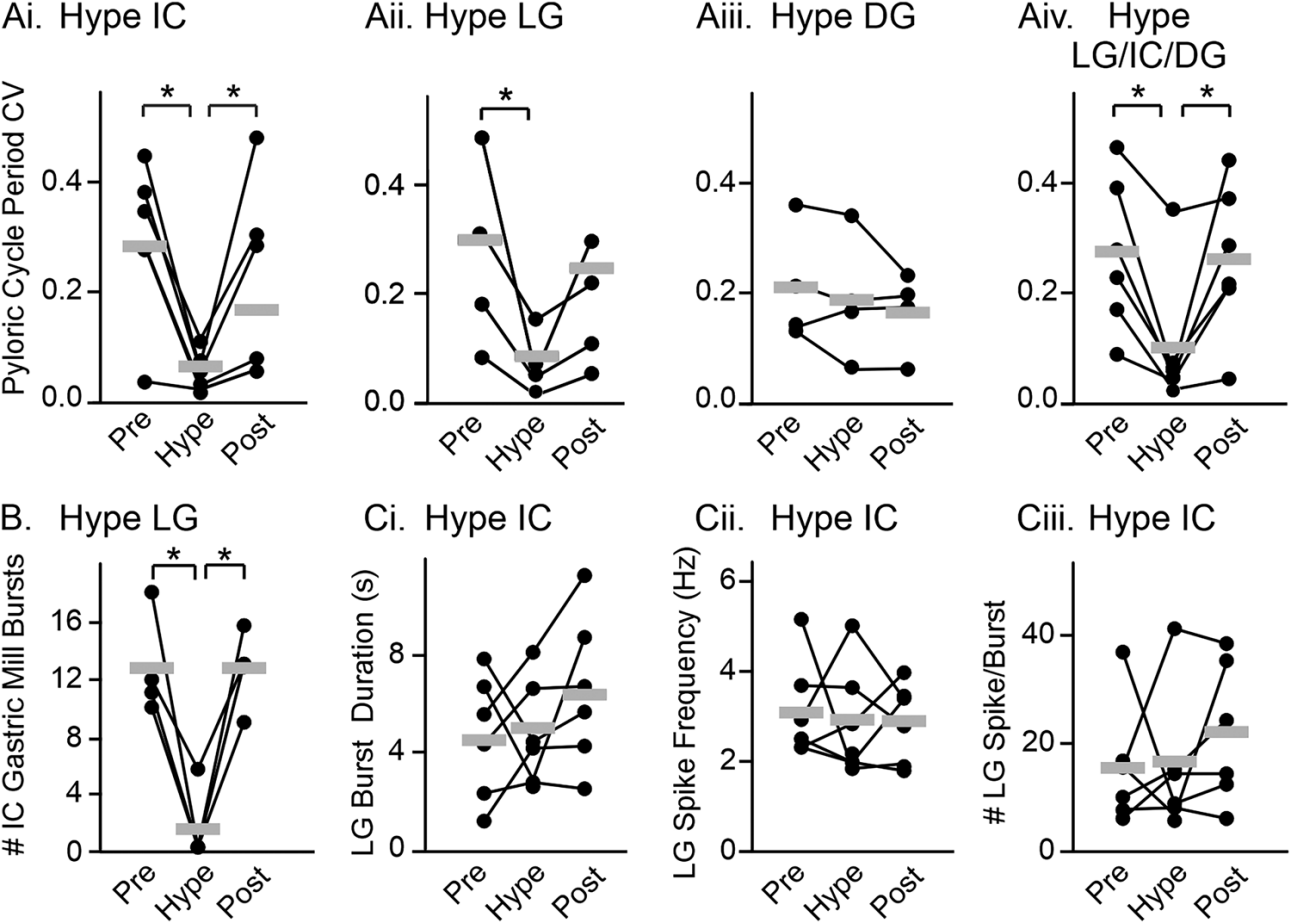
IC hyperpolarization decreased pyloric cycle period variability without altering LG activity. ***A***, The average CV of pyloric cycle period across 200 s windows is plotted for IC (***Ai***, *n* = 6), LG (***Aii***, *n* = 4), DG (***Aiii***, *n* = 4), or all three neurons (***Aiv***, *n* = 6) before (Pre), during (Hype), and after (Post) hyperpolarization. ***B***, The number of IC gastric mill bursts (burst duration >0.45 s) occurring before (Pre), during (Hype), and after (Post) LG hyperpolarization is plotted (*n* = 4). ***C***, LG burst duration (***Ci***), firing frequency (***Cii***), and number of spikes per burst (***Civ***) before (Pre), during (Hype), and after (Post) IC hyperpolarization are plotted (*n* = 6). For all graphs, data points represent individual experiments connected by lines across conditions. Gray bars indicate the average across experiments. *p < 0.05, one-way RM ANOVA, Holm–Sidak *post hoc* test.

To quantify the results of hyperpolarizing IC and LG, we measured pyloric cycle period CV before (Pre), during (Hype), and after (Post) hyperpolarization (Fig. 4*A*). The pyloric cycle period CV decreased when IC was hyperpolarized (Fig. 4*A*i; black dots and connecting lines indicate results from individual experiments, gray bars indicate averages across experiments; one-way RM ANOVA; *F*_(5,2)_ = 7.43; *p* = 0.01; Table 3) and when LG was hyperpolarized (Fig. 4*Aii*; one-way RM ANOVA; *F*_(3,2)_ = 6.09; *p* = 0.04; Table 3). We also determined that the pyloric cycle period CV was not altered when DG was hyperpolarized (Fig. 4*Aiii*; one-way RM ANOVA; *F*_(3,2)_ = 1.18; *p* = 0.37; Table 3) but it was lower when all three gastric mill neurons (IC, LG, and DG) were hyperpolarized (Fig. 4*Aiv*; one-way RM ANOVA; *F*_(5,2)_ = 9.13; *p* = 0.01; Table 3). In the cumulative data, a lower variability when IC or LG or the three gastric mill neurons were hyperpolarized further indicated that IC and/or LG, but not DG, were responsible for regulating pyloric cycle period. When LG was hyperpolarized, IC gastric mill bursting was eliminated in the example experiment in Figure 3*B*. Therefore, we aimed to quantify IC and LG activity across all experiments when either LG or IC was hyperpolarized. However, in 3/4 experiments, there were no IC gastric mill bursts when LG was hyperpolarized. Therefore, instead of quantifying IC gastric mill bursts, we reported the number of IC gastric mill bursts before (Pre), during (Hype), and after (Post) LG hyperpolarization (Fig. 4*B*). There was a reversible decrease in IC gastric mill bursts with LG hyperpolarization (Fig. 4*B*; one-way RM ANOVA; *F*_(3,2)_ = 22.75; *p* = 0.002; Table 3). In contrast, when IC was hyperpolarized, LG activity, including burst duration (Fig. 4*Ci*), firing frequency (Fig. 4*Cii*), and number of spikes per burst (Fig. 4*Ciii*), did not change (one-way RM ANOVA; *F*_(5,2)_ = 0.04–1.52; all conditions, *p* > 0.05; Table 3). Thus, LG impacts IC activity, but IC does not alter LG activity. It therefore appears likely that hyperpolarizing LG eliminated extended pyloric cycle periods via elimination of IC gastric mill bursting. In combination with a significant contribution of LG to pyloric cycle period variability, only when IC is active (above) these results indicate that IC, but not LG, gastric mill-timed bursts are responsible for the longer pyloric cycle periods.

**Table 3.** Pyloric cycle period CV and IC and LG gastric mill activity in Gly^1^-SIFamide (5 μM) before, during, and after hyperpolarization of gastric mill neurons

Manipulation	Parameter	Mean ± SEM			*F* (df)	*p* value		
		Pre	Hype	Post		Pre vs Hype	Hype vs Post	Pre vs Post
Hype IC (*n* = 6)	Pyloric cycle period CV	0.30 ± 0.06	0.09 ± 0.02	0.25 ± 0.07	7.43 (5,2)	0.01	0.04	0.39
Hype LG (*n* = 4)		0.28 ± 0.09	0.06 ± 0.02	0.17 ± 40.06	6.09 (3,2)	0.04	0.15	0.22
Hype DG (*n* = 4)		0.21 ± 0.05	0.19 ± 0.06	0.17 ± 0.04	1.18 (3,2)	ns	ns	ns
Hype IC/LG/DG (*n* = 6)		0.27 ± 0.066	0.10 ± 0.05	0.26 ± 0.06	9.13 (5,2)	0.01	0.01	0.86
Hype LG (*n* = 4)	# IC bursts	12.75 ± 1.80	1.50 ± 1.50	12.75 ± 1.44	22.75 (3,2)	0.003	0.002	1
Hype IC (*n* = 6)	LG burst duration (s)	4.63 ± 1.01	5.08 ± 0.95	6.51 ± 1.25	1.52 (5,2)	ns	ns	ns
	LG # spikes/burst	15.34 ± 5.16	16.50 ± 5.54	21.79 ± 5.73	0.49 (5,2)	ns	ns	ns
	LG firing Freq. (Hz)	2.90 ± 0.39	2.91 ± 0.49	3.07 ± 0.46	0.04 (5,2)	ns	ns	ns

Pre, before hyperpolarization of neuron(s); Hype, during hyperpolarization of neuron(s); Post, after hyperpolarization of neuron(s); CV, coefficient of variation; df, degrees of freedom; ns, p*ost hoc* not performed when *p* > 0.05; one-way repeated measures ANOVA, Holm–Sidak *post hoc.*

### LPG contributes to pyloric cycle period variability in Gly^1^-SIFamide

**Figure 5.**
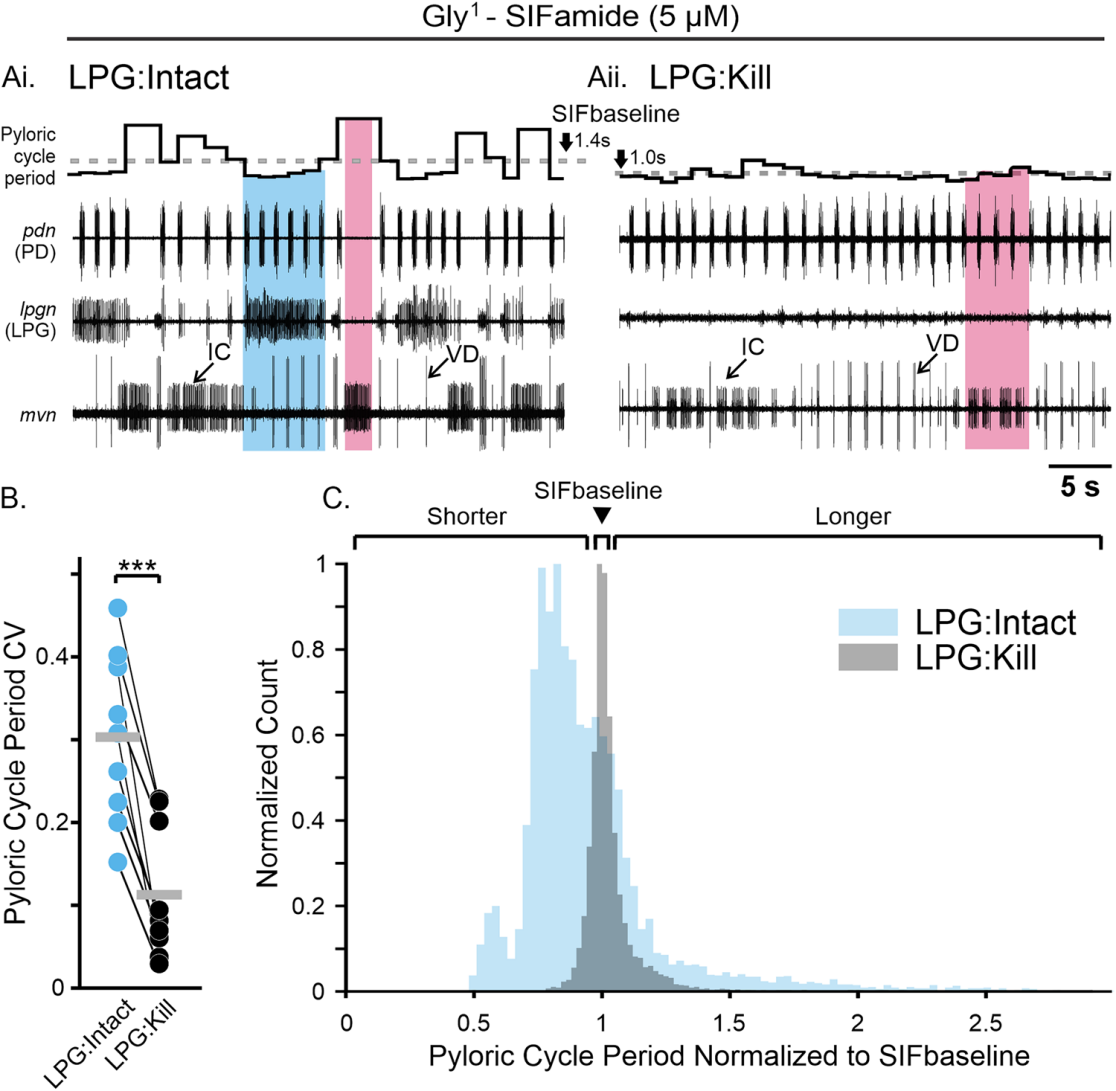
LPG photoinactivation decreased pyloric cycle period variability in Gly^1^-SIFamide. ***Ai***, In Gly^1^-SIFamide with LPG neurons intact, there are shorter cycle periods during LPG gastric mill-timed bursts (blue box), compared with SIFbaseline (dashed line) and longer cycle periods during IC activity (pink box). ***Aii***, After both LPG neurons were photoinactivated (see Materials and Methods), pyloric cycle period variability appeared lower. Pink box highlights an IC gastric mill burst (group of pyloric-timed bursts >0.45 s duration). The small units in the *lpgn* recording are PY neurons. ***B***, The pyloric cycle period CV is plotted for a 20 min window of steady-state activity in the LPG:Intact (blue dots) and LPG:Kill (black dots) conditions (*n* = 9). Each pair of dots plus their connecting lines represents a single preparation. Gray bars indicate the average CV across experiments in each condition. ***p < 0.001, paired *t* test. ***C***, Overlaid histograms (bin size: 3 ms) of all pyloric cycle periods across 20 min windows during steady-state Gly^1^-SIFamide application in the LPG:Intact (blue) and LPG:Kill (gray) conditions (*n* = 9). Each individual cycle period was normalized to the average SIFbaseline of the same preparation. Bin count of each condition was normalized to the peak count of that condition to facilitate comparison of histograms. Brackets on top of histogram indicate shorter pyloric cycle periods, SIFbaseline (arrow), and longer pyloric cycle periods.

LPG gastric mill-timed activity correlated with decreased pyloric cycle periods (Figs. 1, 2, 5). LPG is electrically coupled to the other pyloric pacemaker neurons (Fig. 1*B*; Marder and Bucher, 2007; Shruti et al., 2014), and thus hyperpolarizing current injection into the two LPG neurons could alter the pyloric cycle period. Therefore, to test whether LPG was responsible for decreased pyloric cycle periods relative to SIFbaseline (Fig. 5*Ai*), the two LPG neurons were photoinactivated (see Materials and Methods; LPG:Kill) to selectively remove them from the network, and pyloric cycle periods during Gly^1^-SIFamide bath application were compared between LPG:Intact and LPG:Kill. Photoinactivation of two neurons does not alter the response to bath-applied Gly^1^-SIFamide, and responses to two consecutive bath applications of Gly^1^-SIFamide are not different (Fahoum and Blitz, 2024).

In the absence of the LPG neurons (LPG:Kill), the pyloric cycle period variability decreased (Fig. 5*Aii*) compared with the LPG:Intact condition. Eliminating LPG prevented the periodic decreases in pyloric cycle period. Across experiments, the pyloric cycle period CV was lower after LPG:Kill, compared with LPG:Intact [Fig. 5*B*; LPG:Intact (blue dots) CV = 0.30 ± 0.03; LPG:Kill (black dots) CV = 0.11 ± 0.03; *n* = 9; two-tailed paired *t* test; *t*_(8)_ = 7.78; *p* < 0.001]. However, the decreased variability did not appear to be due to only elimination of shorter pyloric cycle periods. The longer duration pyloric cycle periods were also mostly eliminated in the LPG:Kill condition as can be seen in the example experiment (Fig. 5*Aii*). Assessing regulation of pyloric cycle period across preparations was complicated by the absence of LPG which eliminated several combinations of active gastric mill neurons. Thus, we chose instead to examine the distribution of cycle periods in the LPG:Intact versus LPG:Kill conditions. There is variability in SIFbaseline pyloric cycle period across preparations (Fig. 2*B*, gray box), and photoinactivation of the two LPG neurons shifted the SIFbaseline to shorter pyloric cycle periods (compare instantaneous pyloric cycle period plot in Fig. 5*Ai*–*ii*), likely due to removal of “electrical drag” on the pyloric pacemaker neuron AB (Kepler et al., 1990). Thus, to determine whether there was any change in pyloric cycle period regulation between the two conditions, we normalized each pyloric cycle period to the average SIFbaseline cycle period in that preparation and accumulated all normalized pyloric cycle period durations during each condition into their respective histograms (Fig. 5*C*; LPG:Intact, blue and LPG:Kill, gray). We also normalized pyloric cycle period count per bin to the highest count for each condition to better enable comparison of the histograms. In the LPG:Intact condition, in addition to the peak around 1 (SIFbaseline, arrowhead), there were shorter pyloric cycle periods (<1; “Shorter” bracket) and a distributed set of bins at longer cycle periods (>1; “Longer” bracket; Fig. 5*C*, blue histogram). However, in the LPG:Kill condition (Fig. 5*C*, gray histogram), there was a single grouping of bars centered around 1 (SIFbaseline) without a population of slower or faster cycle periods. Therefore, removing LPG from the network prevented the increase and the decrease in pyloric cycle period. Electrical coupling between LPG and the pyloric pacemaker can explain why the pyloric rhythm was faster during the strong depolarizations of LPG slow bursts. However, this does not explain why removing LPG also eliminated slowing of the pyloric rhythm. Further, we showed above that IC gastric mill bursting was responsible for extending the pyloric cycle period, slowing the rhythm (Figs. 3, 4). This combination of findings led us to ask whether photoinactivation of LPG alters IC gastric mill activity.

### IC gastric mill bursts were not impacted by LPG photoinactivation

**Figure 6.**
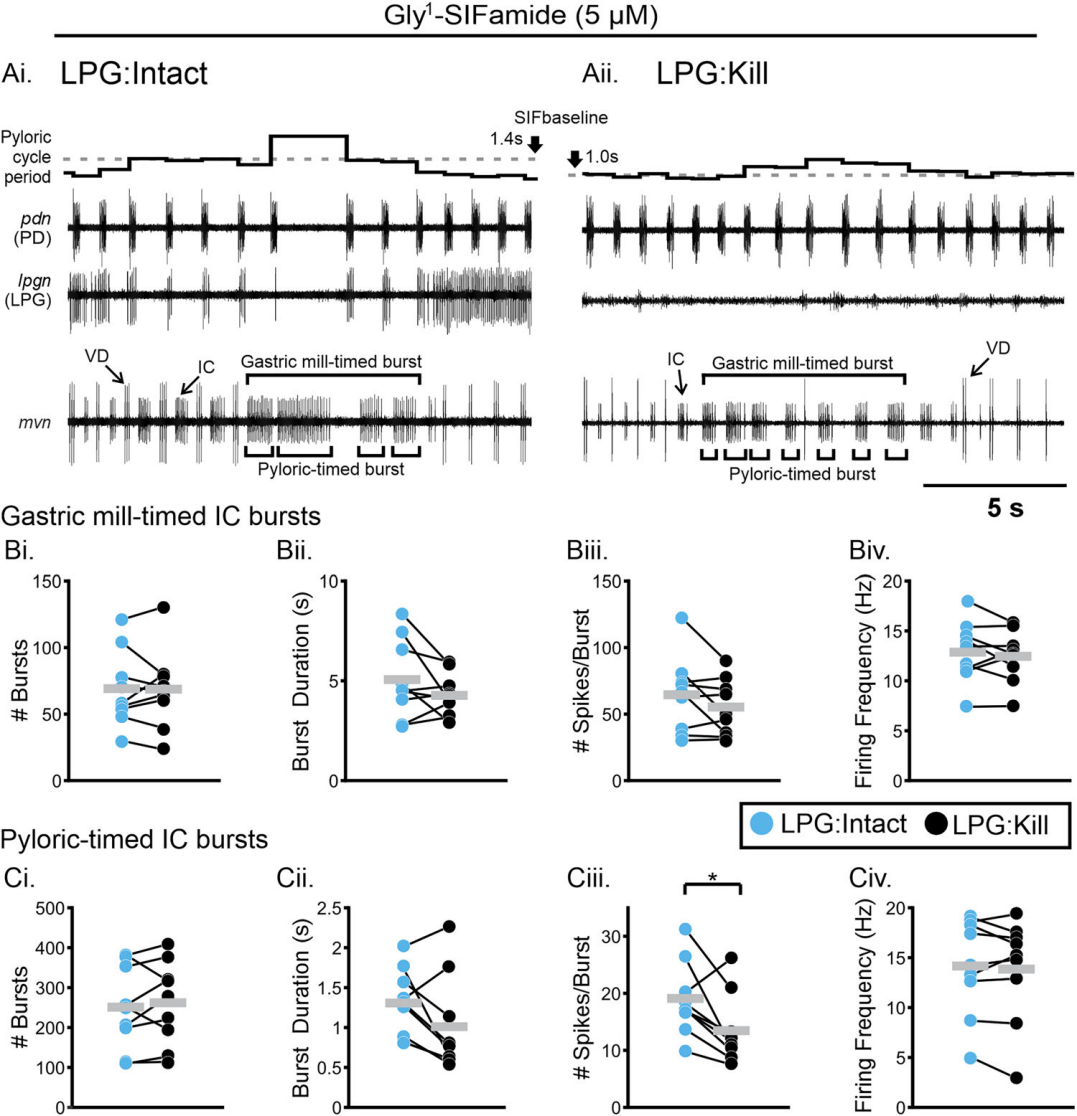
IC firing frequency was not affected by LPG photoinactivation. ***A***, Representative traces illustrate differences in IC gastric mill-timed activity (brackets on top of *mvn* recording) before (***Ai***, LPG:Intact) and after (***Aii***, LPG:Kill) photoinactivation of the two LPG neurons. Pyloric-timed IC bursts within each IC gastric mill burst were identified if there was a gap between IC action potentials that coincided with a PD neuron burst (brackets below *mvn* recording). ***B***, IC gastric mill-timed activity was quantified across 20 min windows during steady-state Gly^1^-SIFamide bath application, including number of bursts (***Bi***), burst duration (***Bii***), number of spikes/burst (***Biii***), and firing frequency (***Biv***). ***C***, IC pyloric-timed activity within each gastric mill-timed burst was also quantified, including number of bursts (***Ci***) burst duration (***Cii***), number of spikes/burst (***Ciii***), and firing frequency (***Civ***). Blue dots indicate LPG:Intact condition and black dots indicate LPG:Kill condition. All dots represent the average within an experiment with lines connecting the two conditions in each experiment. Gray bars indicate averages across experiments. *p < 0.05, paired *t* test.

As discussed previously, IC displays both pyloric- and gastric mill-timed bursts, with a gastric mill burst often consisting of multiple pyloric-timed bursts (Fig. 6*A*). In an example expanded region, a single IC gastric mill burst (Fig. 6*Ai*, brackets above *mvn* recording) in the LPG:Intact condition consists of four pyloric-timed bursts (Fig. 6*Ai*, brackets below *mvn* recording). In the LPG:Kill condition, based on established criteria for IC gastric mill bursts (Blitz et al., 2019; Fahoum and Blitz, 2024), a single IC gastric mill burst is approximately the same duration as in the LPG:Intact condition but consists of seven pyloric bursts, compared with four with LPG neurons intact (Fig. 6*A*). In a previous study, there was no difference in IC activity between LPG:Intact and LPG:Kill, including no difference in burst duration, firing frequency, and number of spikes per burst for gastric mill bursts and for IC pyloric bursts within IC gastric mill bursts (Fahoum and Blitz, 2024). Because our LPG:Intact, LPG:kill dataset was a subset from the previous study (*n* = 9/11, see Materials and Methods), we reanalyzed the data to verify the result in the specific experiments used here. We found no difference in number of IC gastric mill bursts (Fig. 6*Bi*; Table 4), IC gastric mill burst duration (s; Fig. 6*Bii*; Table 4), number of IC spikes per gastric mill burst (Fig. 6*Biii*; Table 4), or firing frequency (Hz; Fig. 6*Biv*; Table 4). There was also no difference in the number of IC pyloric bursts (Fig. 6*Ci*; Table 4), IC pyloric burst duration (s; Fig. 6*Cii*; Table 4), or IC pyloric firing frequency (Hz; Fig. 6*Civ*; Table 4), although there was a trend toward shorter pyloric-timed burst duration (Fig. 6*Cii*; Table 4), and there was a decrease in the number of spikes per pyloric-timed burst (Fig. 6*Ciii*; Table 4). The difference between these results and the previous study likely reflects the removal of two preparations in which there was little pyloric regulation in the control condition. For the nine preparations in which there was typical pyloric regulation in control, the tendency for IC pyloric bursts to be shorter, with fewer action potentials in the LPG:Kill condition, fits with the loss of long-duration pyloric cycle periods. When longer pyloric cycle periods are eliminated, IC neuron pyloric bursts are shortened as they are interrupted by inhibition from the pyloric pacemaker ensemble at the start of each pyloric cycle (Fig. 6*A*). Importantly however, IC gastric mill bursts were not altered by LPG:Kill, and IC firing frequency, a measure of the strength of neuronal activity, was not altered for IC gastric mill or pyloric bursts (Fig. 6*Biv*,*Civ*).

**Table 4.** IC gastric mill- and pyloric-timed activity during LPG Intact and LPG photoinactivated (Killed) conditions in Gly^1^-SIFamide (5 µM)

	Mean ± SEM			
	LPG intact	LPG killed	*t*, df	*p* value
IC gastric mill bursts (*n* = 9)				
# bursts	68.89 ± 9.50	68.33 ± 9.98	0.12, 8	0.91
Burst duration (s)	5.08 ± 0.65	4.33 ± 0.34	1.48, 8	0.18
# Spikes/burst	64.59 ± 9.53	55.02 ± 7.04	1.71, 8	0.13
Firing freq. (Hz)	12.96 ± 1.02	12.47 ± 0.86	0.93, 8	0.37
IC, pyloric bursts (*n* = 9)				
# bursts	249.56 ± 35.09	262.22 ± 35.02	−0.78, 8	0.46
Burst duration (s)	1.31 ± 0.14	1.01 ± 0.20	2.00, 8	0.08
# Spikes/burst	19.02 ± 2.16	13.37 ± 2.03	2.81, 8	0.02
Firing freq. (Hz)	14.14 ± 1.61	13.83 ± 1.72	0.65, 8	0.53

During Gly^1^-SIFamide application; paired *t* test.

### Same IC activity level does not slow the pyloric rhythm in the LPG:Kill condition

**Figure 7.**
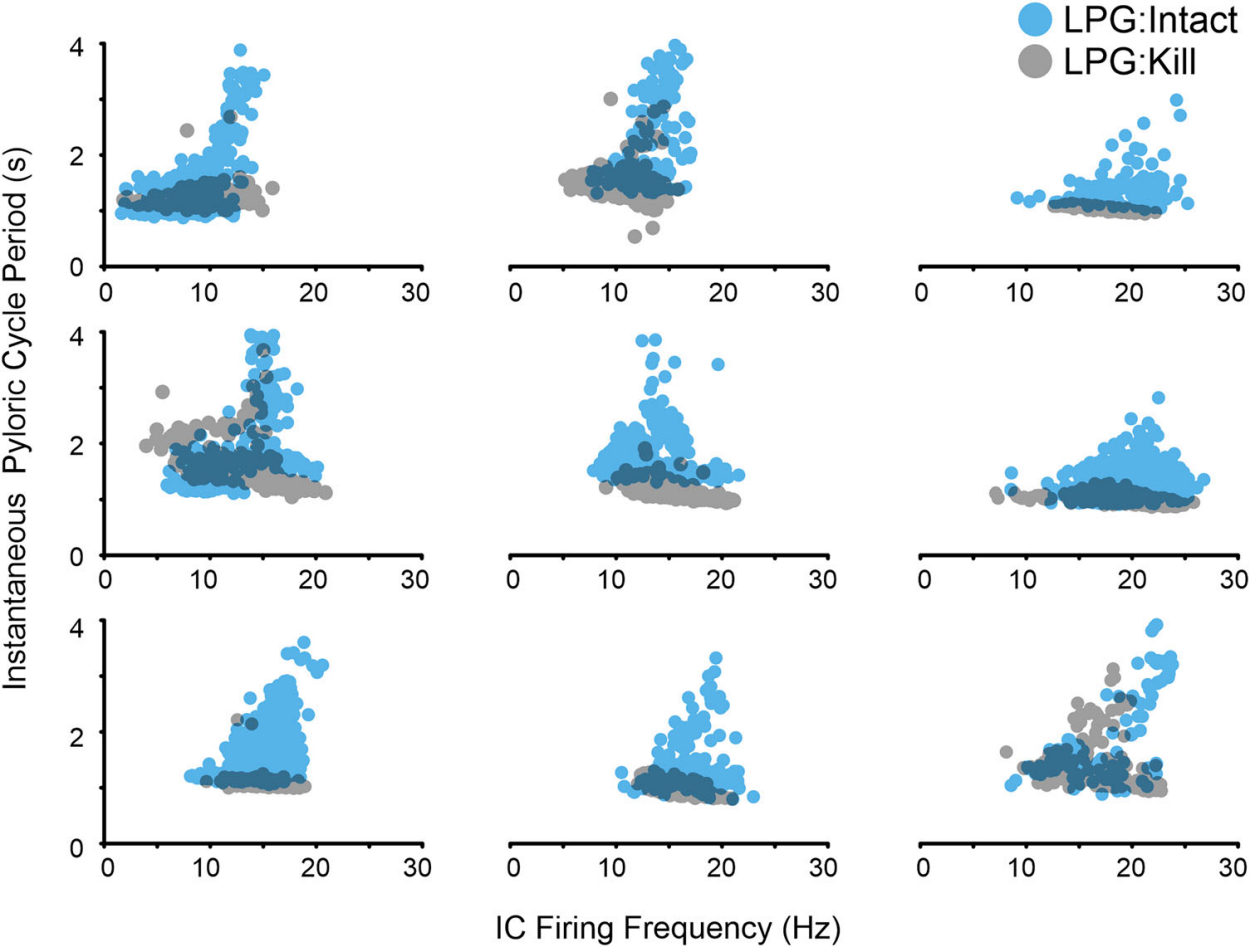
The same IC firing frequency did not alter pyloric cycle period in the absence of LPG neurons. Instantaneous pyloric cycle period is plotted against IC firing frequency in the LPG:Intact (blue dots) and LPG:Kill (gray dots) during 20 min windows. Each graph plots the data from a single experiment and each dot represents the instantaneous pyloric cycle period and the IC firing frequency within its pyloric-timed burst during the same pyloric cycle. Only cycle periods overlapping with IC gastric mill bursts (IC bursts >0.45 s; Fig. 6) are plotted.

**Figure 8.**
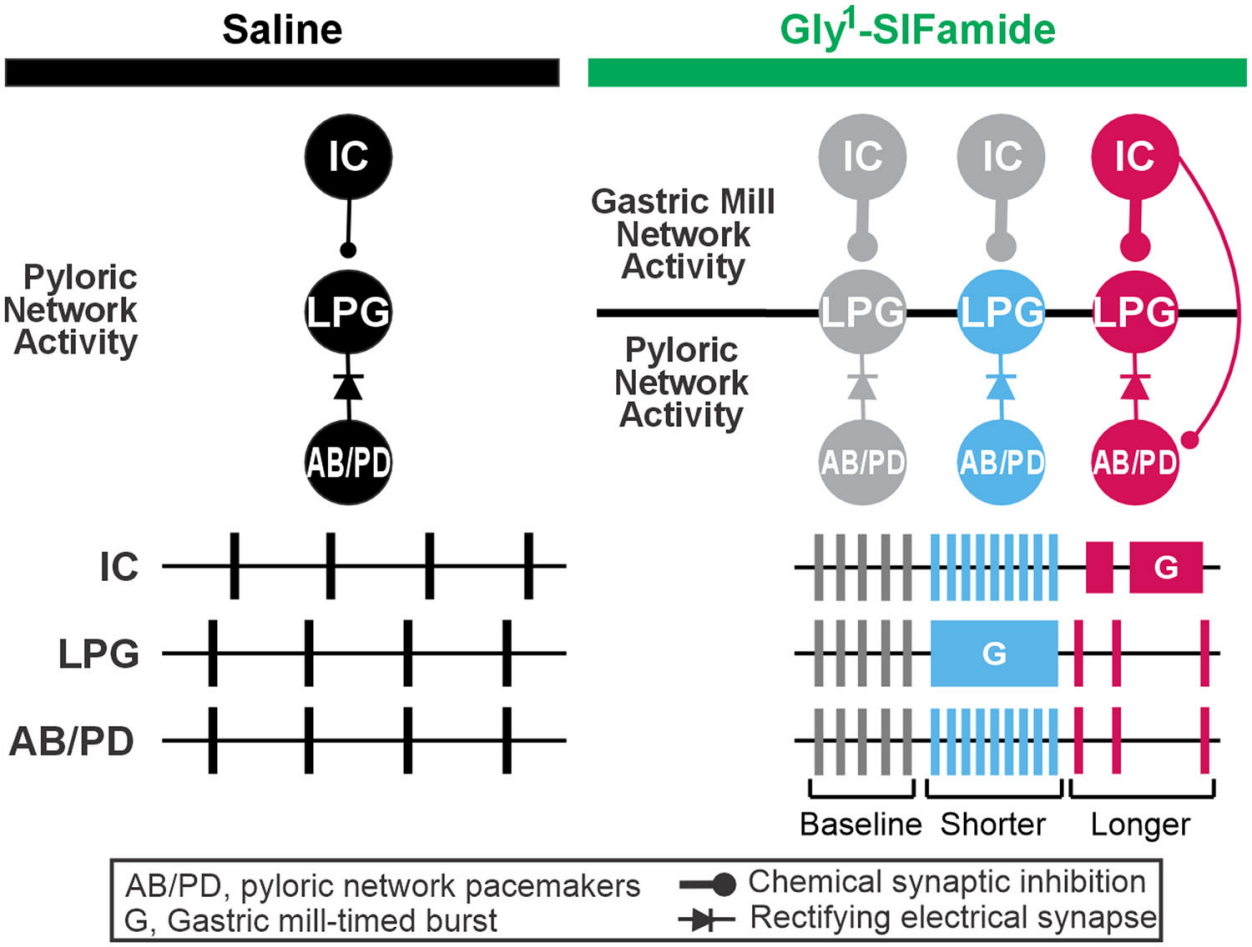
During the Gly^1^-SIFamide modulatory state, gastric mill bursting in LPG decreases pyloric cycle period, while gastric mill bursting in the IC neuron requires LPG to prolong the pyloric cycle period. In saline (left, black traces and circuitry), LPG is active only in pyloric time, coincident with the AB/PD pyloric pacemaker neurons. Gly^1^-SIFamide (right, green bar), elicits a unique gastric mill rhythm (Blitz et al., 2019; Fahoum and Blitz 2021, 2024), during which the pyloric cycle period varies between a baseline level due to Gly^1^-SIFamide modulation (gray), shorter periods which occur during LPG gastric mill-timed slow bursting (blue), and longer periods which occur during IC gastric mill bursting (pink). Gastric mill bursting in LPG (blue box, G, gastric mill burst) likely allows sufficient current though the rectifying electrical synapses (diode symbol) to the AB and PD neurons to decrease the cycle period of the pyloric rhythm (blue). IC gastric mill bursting (pink burst; G, gastric mill burst) is necessary for rhythmic increases in cycle period of the pyloric rhythm (pink). However, this inhibition appears to act primarily via IC chemical synaptic inhibition of LPG (thick pink stick/ball), with a possible weak contribution via chemical inhibition of the AB and/or PD neuron (thin pink line/ball). The IC→LPG inhibitory synapse is strengthened during Gly^1^-SIFamide modulation (compare thin line/ball in saline with thick line/ball in Gly^1^-SIFamide; Fahoum and Blitz, 2023).

To determine the relationship between the strength (firing frequency) of IC gastric mill bursts and pyloric cycle period before versus after LPG:Kill, we plotted instantaneous pyloric cycle period against IC firing frequency (Hz) for all pyloric cycles across a 20 min steady-state region of Gly^1^-SIFamide application for each of nine preparations (Fig. 7). Within a preparation, across the same IC firing frequencies, there were many longer duration pyloric cycle periods in LPG:Intact (blue circles) but many fewer longer duration cycles in the LPG:Kill condition (gray circles; Fig. 7; *n* = 9). This indicated that LPG was playing a major role in the ability of IC to increase pyloric cycle period. The occasional ability of IC to increase pyloric cycle period in the absence of LPG indicated a weaker, or only occasionally modulated, connection to the other pyloric pacemaker neurons (PD and AB; Figs. 7, 8). These data thus suggest that while IC activity strength is not affected by LPG photoinactivation, its ability to slow the pyloric rhythm requires the presence of LPG.

## Discussion

In this study, we show that the cycle period of the faster pyloric rhythm varies between three levels in time with different phases of the related, slower gastric mill rhythm during the Gly^1^-SIFamide neuropeptide modulatory state. The LPG neuron switches from pyloric-only activity to dual pyloric and gastric mill activity during Gly^1^-SIFamide application (Fahoum and Blitz, 2021, 2024; Snyder and Blitz, 2022). Surprisingly, we found that this dual-network LPG neuron is necessary for both phasic increases and phasic decreases in pyloric cycle period during this gastric mill rhythm version. Specifically, the pyloric cycle period decreases during the longer duration, intrinsically generated gastric mill-timed bursts in LPG, and the pyloric cycle period is prolonged during IC neuron gastric mill bursts which coincide with a silent period in LPG. The complex biphasic regulation of a related network we identify here is distinct in both form and mechanism from previous examples of internetwork coordination in the STNS during other modulatory states, emphasizing the flexible nature of such coordination.

### Dual-network neuron is necessary for biphasic regulation

There are multiple ways in which coordination occurs between different behaviors and between their underlying CPG networks. Here we focused on a single modulatory state and how the activity of one network varies across a single cycle of a second network’s activity. Unlike other gastric mill rhythm versions, the Gly^1^-SIFamide gastric mill rhythm is triphasic (Blitz et al., 2019; Fahoum and Blitz, 2024). During this triphasic gastric mill rhythm, there is a “baseline” pyloric cycle period due to Gly^1^-SIFamide modulatory actions, and the pyloric rhythm is regulated away from this baseline in opposing directions during different phases of the slower gastric mill rhythm.

Photoinactivation to selectively eliminate the LPG neurons demonstrated that LPG is necessary for the phasic decreases in pyloric cycle period during the Gly^1^-SIFamide gastric mill rhythm. Decreased pyloric cycle periods occur during LPG gastric mill-timed bursts, which are intrinsically generated. In particular, Gly^1^-SIFamide enhances excitability and postinhibitory rebound and diminishes spike-frequency adaptation in LPG, enabling LPG to generate gastric mill-timed oscillations that do not require any synaptic input (Fahoum and Blitz, 2021; Snyder and Blitz, 2022). An LPG gastric mill-timed burst consists of a sustained depolarization during which LPG intrinsic currents apparently enable it to “escape” the influence of rhythmic hyperpolarizations occurring in its electrically coupled partners, the AB and PD neurons (Shruti et al., 2014; Blitz et al., 2019; Fahoum and Blitz, 2021; Snyder and Blitz, 2022). Electrical synapses are subject to modulation (Kothmann et al., 2009; O’Brien, 2014; Lane et al., 2018). Thus, it is possible that Gly^1^-SIFamide decreases the strength of electrical coupling between LPG and AB/PD to enable the periodic “escapes.” However, the electrical coupling apparently remains functional in Gly^1^-SIFamide, as AB/PD pyloric activity is necessary for LPG to generate pyloric bursting in this state (Fahoum and Blitz, 2021; Snyder and Blitz, 2022). Further, there is no apparent difference in coupling strength between saline and Gly^1^-SIFamide (Fahoum, Nadim, Blitz, unpublished). In addition to the continued presence of electrical coupling between LPG and AB/PD, we know that (1) decreases in pyloric cycle period require the presence of LPG (this study), (2) no other gastric mill neurons are active during LPG gastric mill bursts (Blitz et al., 2019; Fahoum and Blitz, 2024), and (3) LP and IC provide the only known intra-STG feedback to the pyloric pacemakers (Thirumalai et al., 2006; Marder and Bucher, 2007; Nadim et al., 2011; Zhao et al., 2011), but LP is photoinactivated or hyperpolarized and IC is silent or weakly active in pyloric time during this phase (Blitz et al., 2019; Fahoum and Blitz, 2024). Thus, although we are not able to selectively manipulate the electrical synapses between LPG and AB/PD to test our hypothesis, all available evidence indicates that the sustained depolarization during LPG gastric mill bursts has an excitatory effect on the AB/PD neurons to decrease the cycle period of the intrinsic pyloric bursting. This influence occurs despite rectification of the electrical coupling, which favors depolarizing current from AB/PD to LPG, but does allow for some current flow when LPG is depolarized relative to AB/PD (Shruti et al., 2014), which is apparently sufficient to decrease the pyloric cycle period.

Unexpectedly, photoinactivation of the LPG neurons also eliminated increases in pyloric cycle period which occur during IC or IC/LG bursting. With LPG intact, we found that LG promotes gastric mill-timed bursting in the IC neuron, but only IC, and not LG, is necessary for the extended pyloric cycle periods. The potential mechanisms for IC-elicited extension of the pyloric cycle period are that gastric mill-timed IC activity directly inhibits some or all of the pyloric pacemaker neurons or IC gastric mill bursting excites another neuron that inhibits some or all of the pyloric pacemaker neurons. In the Gly^1^-SIFamide gastric mill rhythm, the only neuron besides LG that could be coactive with IC is the LP neuron, which could be “excited” via electrical coupling to IC and does provide inhibitory feedback to the pyloric pacemaker neurons (Weimann and Marder, 1994; Thirumalai et al., 2006; Nadim et al., 2011). However, in our experiments, to mimic the more physiological conditions of Gly^1^-SIFamide release from MCN5, with the coincident inhibition of LP via the MCN5 cotransmitter glutamate (Fahoum and Blitz, 2021), LP was always photoinactivated or hyperpolarized and therefore could not be inhibiting the pyloric pacemaker neurons. Thus, it is most likely that IC extends pyloric cycle period duration by inhibiting pyloric pacemaker neurons, which include one copy of AB and two copies each of PD and LPG.

In many modulatory conditions, the AB neuron is a conditional oscillator, and the PD and LPG neurons are active in pyloric time due to electrical coupling to AB (Ayali and Harris-Warrick, 1999; Marder and Bucher, 2007). In Gly^1^-SIFamide, LPG only generates pyloric-timed bursting if AB/PD do so, but AB/PD do not require LPG to generate pyloric-timed bursting (this study; Fahoum and Blitz, 2021, 2024; Snyder and Blitz, 2022). We found that selective photoinactivation of just the two LPG neurons almost entirely eliminated prolonged pyloric cycle periods. One possible explanation for this was that eliminating LPG impacted the activity of IC; however, we found no change in IC gastric mill bursts. These results suggest that an IC→LPG synapse is primarily responsible for IC inhibition of the pyloric rhythm, effectively “funneling” IC chemical inhibition through LPG’s electrical coupling to the AB/PD neurons (Fig. 8). In fact, we recently determined that Gly^1^-SIFamide enhances a typically ineffective IC→LPG graded glutamatergic synapse, enabling gastric mill IC bursts to regulate LPG gastric mill-timed activity (Fahoum and Blitz, 2023). The current study indicates that the modulated IC→LPG synapse also indirectly regulates the pyloric cycle period via LPG’s electrical coupling to AB/PD. Some prolonged pyloric cycle periods in a subset of preparations when LPG was photoinactivated suggests an additional small contribution of an IC→AB and/or IC→PD synapse; however, it seems that the main IC regulation of pyloric cycle period occurs via inhibition of LPG (Fig. 8). The reason for IC chemical inhibition to act indirectly via the electrical synapse from LPG to AB/PD is not known. However, it may represent a conservation of function with the IC→LPG synapse playing roles in both gastric mill pattern generation as well as coordination between the pyloric and gastric mill networks.

Periodic long-duration IC bursts also inhibit the pyloric rhythm during activation of another modulatory input to the STG, the projection neuron MCN7, which releases the neuropeptide proctolin (Blitz et al., 1999). The specific neuron(s) targeted by IC to extend the pyloric cycle period in the MCN7 rhythm was not determined (Blitz et al., 1999). IC chemical synapses within the STG, including the IC→LPG synapse, are fast inhibitory glutamatergic connections that are blocked by the chloride channel blocker picrotoxin (Marder and Bucher, 2007; Fahoum and Blitz, 2024). Inhibitory neuron connections between networks are implicated in other internetwork interactions, although further exploration is still needed for a mechanistic understanding at the cellular level (Huff et al., 2022).

### Diverse mechanisms of internetwork coordination

Distinct mechanisms have been identified for internetwork coordination in the STNS and other systems. In the STNS, other gastric mill rhythm versions are biphasic, and when pyloric rhythm variability has been quantified, the pyloric period during one phase of the gastric mill rhythm is at a “baseline” period due to modulatory input and regulated away from this baseline during the other phase of the gastric mill rhythm (Bartos and Nusbaum, 1997). For instance, during a gastric mill rhythm elicited by the modulatory neuron MCN1, the LG neuron presynaptically inhibits MCN1 at its entrance into the STG, rhythmically decreasing modulatory excitation of the pyloric network during the LG phase (Nusbaum et al., 1992; Coleman and Nusbaum, 1994; Coleman et al., 1995; Bartos and Nusbaum, 1997). Thus, there is indirect communication between the two networks via a local feedback synapse. Indirect interactions between networks can also occur via long-distance feedback from network neurons to modulatory inputs or via motor efference copy to related networks (Wood et al., 2004; Blitz and Nusbaum, 2008; Lambert et al., 2023). Although there are both local and long-distance feedback projections to the source of Gly^1^-SIFamide (MCN5; Norris et al., 1996; Blitz et al., 2019), they do not contribute to the internetwork regulation described here, as the neuropeptide was bath applied. Instead, we found that synapses between network neurons underlie biphasic regulation of the pyloric rhythm.

In addition to phasic coordination, there may be correlated changes in the activity levels of related networks such as increased respiratory activity to match more intense forms of locomotion or increased swallowing occurrences to match increased chewing rates (Hao et al., 2021; Hérent et al., 2023). When the pyloric and gastric mill networks are both active, their activity level is coordinated such that faster pyloric rhythms occur with faster gastric mill rhythms (Bartos et al., 1999; Saideman et al., 2007; Powell et al., 2021). Correlated changes in timing and/or strength can reflect common inputs. In the crab STNS, the modulatory neuron MCN1 acts on both networks, and increasing MCN1 firing frequency speeds both rhythms (Bartos and Nusbaum, 1997; Bartos et al., 1999). Similarly, in lamprey, rodents, and cats, the mesencephalic locomotor region provides parallel input to respiratory and locomotor CPGs (Ryczko and Dubuc, 2013; Juvin et al., 2022; Hérent et al., 2023).

Some related behaviors display coupling of their activity, with a discrete number of cycles of one pattern occurring relative to a full cycle of the other pattern. Coupling occurs between locomotion and chewing, and locomotion and respiration in humans, other mammals, and birds; between whisking and respiration in rodents; and between pyloric and gastric mill rhythms in crustaceans (Clemens et al., 1998a; Bartos et al., 1999; Moore et al., 2013; Maezawa et al., 2020; Powell et al., 2021; Juvin et al., 2022). Coupling of rhythmic behaviors may arise from mechanical forces. For example, flight muscle attachment to structures essential for respiration in birds, and the movement of internal organs in galloping horses may contribute to coupling between locomotion and respiration (Juvin et al., 2022). Similarly, accessory teeth movements are mechanically coupled to the cardiopyloric valve in the stomatogastric system (McGaw and Curtis, 2013). However, direct synaptic connections between CPGs can also contribute to this type of internetwork coordination (Juvin et al., 2022). In rodents, proper timing of swallowing within the respiratory cycle, and 1:1 coupling of whisking and fast sniffing, appear to be due to synapses between these related CPGs (Moore et al., 2013; Huff et al., 2022). These coupling ratios between networks are not fixed and can vary during different forms of locomotion, or different breathing behaviors such as fast sniffing and slow breathing (Saunders et al., 2004; Moore et al., 2013; Juvin et al., 2022). In the crab STNS, a direct inhibitory synapse from the pyloric pacemaker neuron AB onto the gastric mill CPG neuron Int1 is essential for coupling between the pyloric and gastric mill rhythms driven by the modulatory neuron MCN1. This internetwork synapse allows the pyloric rhythm to control the speed of the gastric mill rhythm (Nadim et al., 1998; Bartos et al., 1999). However, during the Gly^1^-SIFamide rhythm, the speed of the pyloric rhythm does not regulate LPG gastric mill bursting, reinforcing the flexible nature of internetwork coordination (Fahoum and Blitz, 2024).

Our results here suggest that the same rectifying electrical synapse alternately reinforces and diminishes modulatory neuropeptide effects on network output. These actions occur due to intrinsic currents enabling reinforcement, and chemical synaptic currents leading to diminished peptide actions. LPG thus serves as a point of convergence for coordination, despite the two network interactions acting in opposite directions. Due to the fluid nature of network composition, dual-network neurons such as LPG provide flexibility by adding and subtracting their activity to different behavioral outputs (Hooper and Moulins, 1989; Dickinson et al., 1990; Weimann et al., 1991; Roopun et al., 2008; Ainsworth et al., 2011; Clapp et al., 2011). Additionally, dual-network neurons can provide flexibility in coordinating network activity by serving as a variable link between networks. This ability however depends on whether they are recruited into new networks as passive followers or as active components with sufficient synaptic access to a new network to mediate coordination (Hooper and Moulins, 1990; Fahoum and Blitz, 2024).

Dysfunctional internetwork coordination can impact health and well-being, such as an insufficient oxygen supply relative to metabolic demand if respiration is not coordinated with other motor activity, decreased food intake or choking due to improper coordination of breathing and swallowing, or disrupted communication abilities arising from problems coordinating vocalization and respiration (Barlow, 2009; Yagi et al., 2017; Juvin et al., 2022). Thus, it is important to continue adding to our understanding of internetwork coordination among the many behaviors that require coordination, including understanding how coordination varies across behavioral and environmental conditions (Clemens et al., 1998b; Stickford and Stickford, 2014; Stein and Harzsch, 2021; Juvin et al., 2022).

## Synthesis

Reviewing Editor: Arianna Maffei, Stony Brook University Decisions are customarily a result of the Reviewing Editor and the peer reviewers coming together and discussing their recommendations until a consensus is reached. When revisions are invited, a fact-based synthesis statement explaining their decision and outlining what is needed to prepare a revision will be listed below. The following reviewer(s) agreed to reveal their identity: David Schulz.

The reviewers agree that this study provides a valuable contribution to the field. However, they also agree that the manuscript needs to be revised in order to clarify information and put it in the context of current thinking in the field.

In addition to a few minor points listed in the point by point comments of the reviewers listed below, there are a number of major issues with the manuscript:

1- the graphical abstract is not well organized and does not convey the take home message of the manuscript. It should be redesigned.

2- the discussion is vague, unfocused and not effective at discussing the findings and does not provide a clear context for the interpretation of the data. It should be substantially edited.

3- both reviewers found Figure 8 and its legend particularly dense and confusing. It should be edited to improve clarity.

Additional comments are listed below.

Reviewer #1

Overall the authors have done a quality job presenting clear representative traces and figures to describe the interesting phenomenon on circuit interactions via the LPG neuron among the gastric and pyloric circuits of the STG. These are complex recordings and often hard to grasp, but the data as presented were clear. I agree with the authors' assertion that this provides an interesting look at mechanistic interaction among circuits where few other systems have been able to provide this level of resolution.

I have the following major and minor comments that I would suggest the authors consider in a revision:

The major limitation of this work is the interpretation of the mechanistic interaction working via electrical synapses without direct evidence or experimentation to that fact. The electrical synaptic influence is purely inferential in this case - and built on a long history of data collected in this system (most of it from other studies). The sum total of the new data presented are as described in the opening paragraph of the Discussion - and it is telling that electrical synapses are not mentioned in this opening summary. What is essential to make this conclusion is direct manipulation of the electrical synapses in a focal and specific manner. Rather, the authors have inferred that through cell kills and knowledge of the circuit connectivity that the most likely mechanism involves rectifying electrical synapses. I happen to agree with their interpretation, but it is not unequivocal. That said, I know direct manipulation of these synapses is not possible in this system. Therefore, without the ability to directly implicate electrical synapses, I would recommend that the authors do two things. One, step back a bit from the certainty with which this is mechanistically discussed in this study (including the title). Two, more deliberately and clearly "connect the dots" that led them to this conclusion. A substantive re-writing of the Discussion would accomplish this. I recommend the following specific revisions (in addition to any inspiration the authors have to streamline the presentation and interpretation of their data towards their major inference):

1) The quite long and somewhat vaguely written section that leads the Discussion ("Coordination of Related Behaviors") is fairly distracting and somewhat unnecessary for the data at hand. This could be greatly reduced and moved to the end of the Discussion if it is retained. The most pertinent pieces of this (such as lines 661-677) are somewhat vague. Specifically the sentence that starts on line 665. The reader will benefit from more specifics, including the identities of the neurons described in sentences like these.

2) Provide a less narrative and a more literal step by step through which the reader could achieve this same interpretation, including demonstration as to how the data in this paper are the final piece needed to reach this conclusion. Perhaps even a numbered list with references for each piece of the conclusion would be effective.

3) Because the implication of the electrical synapses is largely inferential, the authors should more overtly and carefully provide alternative explanations and hypotheses - and why they are a less reasonable conclusion to draw.

4) Figure 8 may be more effective if it were split into multiple panels that show under what conditions a given synapse is strengthened, weakened, and the resulting (schematic) impact on output. Perhaps for the three firing modes shown of the pacemaker, three circuit maps could be shown each with the maximally influential synapses highlighted.

5) With all acknowledgment of the difficulty of putting these things together, the graphical abstract is largely inscrutable and would be of little informative (or aesthetic) value to someone glancing at it without reading the paper first. However, it is the leftmost panel of the graphical abstract that hints at my suggestions for edits to Figure 8, with three distinct phases of firing and a clearer highlighting of the key synaptic interactions underlying.

6) Neuromodulation seems to have been lost in the discussion, and it is not clear how the peptide modulator and its known effects on neurophysiological properties of these cells are involved in the mechanistic interpretation - and would be a valuable addition to the "list" proposed in point 2 above.,

7) The title really could use a chance to be clearer with respect to the content of the study, especially as there is no indication in the title that there is a role for different states induced by modulation.

Minor Comments:

Line 474: "and or"

Line 742: awkward....."IC inhibition of LPG....inhibits LPG"

Reviewer #2

This paper describes a well conducted study using standard electrophysiological techniques that determines the cellular bases of the complicated coordination between the gastric and pyloric rhythms during the triphasic Gly1-SIFamide elicited gastric rhythm in the crab stomatogastric nervous system. The STNS is an important experimental system for determining how CPG networks operate at cellular and network level. Indeed, the analysis of this system has had a major impact on how we understand all rhythmic networks. The experiments are carefully performed and analyzed, and the data is clear and convincing. Some of the data comes from previous studies by the same author(s), which I view as a plus; something that leaves published data less isolated and indeed useful. The figures are very easy to comprehend and show the data well. The tables are well tables, but they do the trick. I do have some suggested revisions. None are really major, so I'll just list them together.

Abstract:

I think you should mention that LG enables IC gastric bursting.

Figures:

1. In Figure 1A, do the specific colors indicate anything meaningful?

2. In Figure 1Ciii indicate the three phases of the Gly1-SIFamide elicited gastric rhythm.

3. Figure legend 8 Lines 1168-1171 are very confusing because the pyloric rhythm itself is typically triphasic. I suggest, "Pyloric cycle period durations during the triphasic Gly1-SIFamide elicited gastric rhythm are depicted below with schematized AB/PD activity during SIFbaseline (grey), speeding due to LPG gastric mill-timed slow bursting (blue) and slowing caused by IC gastric mill bursting (pink)."

Introduction:

1. Lines 164-165: '...nesting...nesting...' Eliminate one nest.

2. Introduction could spend more effort on explaining the STNS networks and less on the comparison to other systems.

Methods (very difficult to follow in some places):

1. Parameters. I disagree with the use of "parameters" to describe measured output features.

"TECHNICAL

noun: parameter; plural noun: parameters

a numerical or other measurable factor forming one of a set that defines a system or sets the conditions of its operation.

"the transmission will not let you downshift unless your speed is within the lower gear's parameters"

MATHEMATICS

a quantity whose value is selected for the particular circumstances and in relation to which other variable quantities may be expressed.

STATISTICS

a numerical characteristic of a population, as distinct from a statistic of a sample.

(in general use) a limit or boundary that defines the scope of a particular process or activity.

"they set the parameters of the debate"

In a dynamical system, I think of parameters as values set in model equations that determine the quantitative aspects of the resulting solutions. These are the givens of a system that determine its output. How about output characteristics, measures, properties, features?

2. Line 368 and in Abstract and elsewhere: Why do you speak of frequency when you measure period. It is very confusing. Indeed, Abstract starts the confusion by stating that (gastric mill [chewing, ~0.1 Hz]; pyloric [filtering food, ~1 Hz]). This should be "(gastric mill [chewing, period ~10 s]; pyloric [filtering food, period ~1 s])."

3. Line 419 and elsewhere: I am very confused by this notation. What exactly do you mean by 9-10 and 16-19. Be very specific if you are to use this notation as to what it means.

4. Lines 255-257 and in Results: Please explain why eliminating LP is needed for this 'biological model'.

5. Lines 316-324: This is very hard to follow. Please explain better.

Results:

1. Lines 463-465: Above you state "Further, effects of LPG versus IC, or IC

and LG, appear to offset the impact of the other neuron(s). That is, for example, during

conditions where LPG was active, pyloric cycle periods were shorter than SIFbaseline,

unless IC (or IC and LG) was active (purple regions)." This seems contradictory to me. Can you clarify?

Discussion:

1. This seems overly long and a bit meandering. Reference to other systems and context are all well and good but the reader may give up before reaching the take home at the end.

## Author Response

Dear Dr. Maffei and Reviewers, We thank you for your careful review of our manuscript and your helpful suggestions. We feel the manuscript has been improved and provide our point-by-point responses to all comments below.

Synthesis Statement for Author (Required):

The reviewers agree that this study provides a valuable contribution to the field. However, they also agree that the manuscript needs to be revised in order to clarify information and put it in the context of current thinking in the field.

In addition to a few minor points listed in the point by point comments of the reviewers listed below, there are a number of major issues with the manuscript:

1- the graphical abstract is not well organized and does not convey the take home message of the manuscript. It should be redesigned.

Response: We have used the reviewers' feedback and extensively redesigned the graphical abstract.

2- the discussion is vague, unfocused and not effective at discussing the findings and does not provide a clear context for the interpretation of the data. It should be substantially edited.

Response: The discussion has been substantially revised with an emphasis on the interpretation of the data.

3- both reviewers found Figure 8 and its legend particularly dense and confusing. It should be edited to improve clarity.

Response: Figure 8 has also been substantially revised and the legend re-written accordingly.

Reviewer #1 Overall the authors have done a quality job presenting clear representative traces and figures to describe the interesting phenomenon on circuit interactions via the LPG neuron among the gastric and pyloric circuits of the STG. These are complex recordings and often hard to grasp, but the data as presented were clear. I agree with the authors' assertion that this provides an interesting look at mechanistic interaction among circuits where few other systems have been able to provide this level of resolution.

I have the following major and minor comments that I would suggest the authors consider in a revision:

The major limitation of this work is the interpretation of the mechanistic interaction working via electrical synapses without direct evidence or experimentation to that fact. The electrical synaptic influence is purely inferential in this case - and built on a long history of data collected in this system (most of it from other studies). The sum total of the new data presented are as described in the opening paragraph of the Discussion - and it is telling that electrical synapses are not mentioned in this opening summary. What is essential to make this conclusion is direct manipulation of the electrical synapses in a focal and specific manner. Rather, the authors have inferred that through cell kills and knowledge of the circuit connectivity that the most likely mechanism involves rectifying electrical synapses. I happen to agree with their interpretation, but it is not unequivocal. That said, I know direct manipulation of these synapses is not possible in this system. Therefore, without the ability to directly implicate electrical synapses, I would recommend that the authors do two things. One, step back a bit from the certainty with which this is mechanistically discussed in this study (including the title). Two, more deliberately and clearly "connect the dots" that led them to this conclusion. A substantive re-writing of the Discussion would accomplish this. I recommend the following specific revisions (in addition to any inspiration the authors have to streamline the presentation and interpretation of their data towards their major inference):

1) The quite long and somewhat vaguely written section that leads the Discussion ("Coordination of Related Behaviors") is fairly distracting and somewhat unnecessary for the data at hand. This could be greatly reduced and moved to the end of the Discussion if it is retained. The most pertinent pieces of this (such as lines 661-677) are somewhat vague. Specifically, the sentence that starts on line 665. The reader will benefit from more specifics, including the identities of the neurons described in sentences like these.

Response: The discussion has been substantially revised with an emphasis on interpretation of the data and discussion of how we arrived at our conclusions. The section referred to in this comment was partially eliminated, with the retained portion made more explicit as suggested.

2) Provide a less narrative and a more literal step by step through which the reader could achieve this same interpretation, including demonstration as to how the data in this paper are the final piece needed to reach this conclusion. Perhaps even a numbered list with references for each piece of the conclusion would be effective.

Response: We now explicitly discuss previous studies and how we arrived at our interpretation for the regulation in each direction.

3) Because the implication of the electrical synapses is largely inferential, the authors should more overtly and carefully provide alternative explanations and hypotheses - and why they are a less reasonable conclusion to draw.

Response: In line with the previous comment, the rationale behind the suggested role of the electrical coupling is now more explicitly discussed, as are other possibilities, including why they are unlikely.

4) Figure 8 may be more effective if it were split into multiple panels that show under what conditions a given synapse is strengthened, weakened, and the resulting (schematic) impact on output. Perhaps for the three firing modes shown of the pacemaker, three circuit maps could be shown each with the maximally influential synapses highlighted.

Response: We appreciate the helpful suggestions and used them in redesigning this figure. There is a new version of figure 8 and its associated new legend.

5) With all acknowledgment of the difficulty of putting these things together, the graphical abstract is largely inscrutable and would be of little informative (or aesthetic) value to someone glancing at it without reading the paper first. However, it is the leftmost panel of the graphical abstract that hints at my suggestions for edits to Figure 8, with three distinct phases of firing and a clearer highlighting of the key synaptic interactions underlying.

Response: Thank you for the helpful suggestions, we also feel the new graphical abstract is much clearer and in hindsight recognize the inscrutability of our original version.

6) Neuromodulation seems to have been lost in the discussion, and it is not clear how the peptide modulator and its known effects on neurophysiological properties of these cells are involved in the mechanistic interpretation - and would be a valuable addition to the "list" proposed in point 2 above., Response: We agree that neuromodulation was lost in the original discussion and we have tried to remedy this in the revision. We have also discussed more of the modulation actions in our interpretations in response to some of the comments above.

7) The title really could use a chance to be clearer with respect to the content of the study, especially as there is no indication in the title that there is a role for different states induced by modulation.

Response: Based on this comment and comments above about the role of the electrical synapse, we have revised the title as: "Neuropeptide Modulation Enables Biphasic Inter-network Coordination via a Dual-Network Neuron" Minor Comments:

Line 474: "and or" Response: Fixed Line 742: awkward....."IC inhibition of LPG....inhibits LPG" Response: This statement was changed in the revision of the Discussion. The same point is now made here "These results suggest that an IC to LPG synapse is primarily responsible for IC inhibition of the pyloric rhythm, effectively "funneling" IC chemical inhibition through LPG's electrical coupling to the AB/PD neurons (Fig. 8)."

Reviewer #2 This paper describes a well conducted study using standard electrophysiological techniques that determines the cellular bases of the complicated coordination between the gastric and pyloric rhythms during the triphasic Gly1-SIFamide elicited gastric rhythm in the crab stomatogastric nervous system. The STNS is an important experimental system for determining how CPG networks operate at cellular and network level. Indeed, the analysis of this system has had a major impact on how we understand all rhythmic networks. The experiments are carefully performed and analyzed, and the data is clear and convincing. Some of the data comes from previous studies by the same author(s), which I view as a plus; something that leaves published data less isolated and indeed useful. The figures are very easy to comprehend and show the data well. The tables are well tables, but they do the trick. I do have some suggested revisions. None are really major, so I'll just list them together.

Abstract:

I think you should mention that LG enables IC gastric bursting.

Response: We have added this point as suggested. "Hyperpolarizing current injections demonstrated that although LG bursting enables IC bursts, only gastric mill rhythm bursts in IC are necessary to prolong the pyloric cycle period."

Figures:

1. In Figure 1A, do the specific colors indicate anything meaningful? Response: We eliminated the colors from the circuit diagram.

2. In Figure 1Ciii indicate the three phases of the Gly1-SIFamide elicited gastric rhythm.

Response: The three phases are now indicated in figure 1Ciii and the text updated accordingly. We have retained the main explanation of the three phases in the results section explaining figure 2 to avoid redundancy, but also refer to figure 1Ciii as an additional example of the phasic regulation.

3. Figure legend 8 Lines 1168-1171 are very confusing because the pyloric rhythm itself is typically triphasic. I suggest, "Pyloric cycle period durations during the triphasic Gly1-SIFamide elicited gastric rhythm are depicted below with schematized AB/PD activity during SIFbaseline (grey), speeding due to LPG gastric mill-timed slow bursting (blue) and slowing caused by IC gastric mill bursting (pink)." Response: Figure 8 has been revised and the figure legend re-written. We now refer to the individual phases and avoid the term "triphasic".

Introduction:

1. Lines 164-165: '...nesting...nesting...' Eliminate one nest.

Response: Fixed.

2. Introduction could spend more effort on explaining the STNS networks and less on the comparison to other systems.

Response: As suggested, we condensed the comparisons to other systems, and added a little more information on the STNS networks, trying to achieve a balance between providing context based on other systems, and specific STNS information within the word limit.

Methods (very difficult to follow in some places):

1. Parameters. I disagree with the use of "parameters" to describe measured output features. "TECHNICAL noun: parameter; plural noun: parameters a numerical or other measurable factor forming one of a set that defines a system or sets the conditions of its operation. "the transmission will not let you downshift unless your speed is within the lower gear's parameters" MATHEMATICS a quantity whose value is selected for the particular circumstances and in relation to which other variable quantities may be expressed.

STATISTICS a numerical characteristic of a population, as distinct from a statistic of a sample. (in general use) a limit or boundary that defines the scope of a particular process or activity. "they set the parameters of the debate" In a dynamical system, I think of parameters as values set in model equations that determine the quantitative aspects of the resulting solutions. These are the givens of a system that determine its output. How about output characteristics, measures, properties, features? Response: We eliminated parameters and used activity or bursts, or the names of the output characteristics such as LG burst duration, or otherwise changed the wording to avoid the use of parameter, such as "Neuronal Activity Quantification" instead of "Neuronal Activity Parameters" as a header for that section of the methods.

2. Line 368 and in Abstract and elsewhere: Why do you speak of frequency when you measure period. It is very confusing. Indeed, Abstract starts the confusion by stating that (gastric mill [chewing, ~0.1 Hz]; pyloric [filtering food, ~1 Hz]). This should be "(gastric mill [chewing, period ~10 s]; pyloric [filtering food, period ~1 s])." Response: We have changed frequency to period throughout the manuscript.

3. Line 419 and elsewhere: I am very confused by this notation. What exactly do you mean by 9-10 and 16-19. Be very specific if you are to use this notation as to what it means.

Response: We have revised the way these numbers are discussed. "Across 19 preparations, there were some preparations in which no pyloric cycle periods overlapped with a combination of neurons. Specifically in the IC, LPG:IC, and LPG:DG:IC categories there were between 9 and 10 preparations with no pyloric cycle periods falling into these categories. In the 13 remaining categories there were 0 to 3 preparations that had no pyloric cycles in these categories."

4. Lines 255-257 and in Results: Please explain why eliminating LP is needed for this 'biological model'.

Response: This is now explained in the methods: "For the established biological model of MCN5 actions, it is necessary to eliminate LP activity to mimic the MCN1 glutamatergic inhibition of LP" and in the results: "MCN5 inhibits LP via its cotransmitter glutamate, but Gly1-SIFamide bath application excites LP, likely mimicking the actions of a second Gly1-SIFamide-containing input to the STG". Therefore, we hyperpolarized or photoinactivated the LP neuron to enable LPG dual-network activity and better mimic the MCN5-elicited motor pattern." 5. Lines 316-324: This is very hard to follow. Please explain better.

Response: These are the number of preparations in which there were cycle periods classified as the categories listed. We tried to clarify this by referring to the number of preparations in which there were no pyloric cycles classified within each set of categories, as that was the point raised immediately prior to this sentence. We now state: "In some preparations, no pyloric cycle periods were assigned to some of the categories. Specifically, for IC, LPG:IC, and LPG:DG:IC there were 9 to 10 preparations that had no cycle periods assigned to each of these categories while for the remaining 13 categories there were 0 to 3 preparations that had no pyloric cycles assigned to these categories." (new line 238) Results:

1. Lines 463-465: Above you state "Further, effects of LPG versus IC, or IC and LG, appear to offset the impact of the other neuron(s). That is, for example, during conditions where LPG was active, pyloric cycle periods were shorter than SIFbaseline, unless IC (or IC and LG) was active (purple regions)." This seems contradictory to me. Can you clarify? Response: You are correct, this is contradictory. Our original visual inspection led us to include the indication that LPG and IC (or IC/LG) effects could offset each other, but it was not supported by the statistical analysis. We have removed this statement and removed the purple regions from figure 2. We appreciate the reviewer taking note of this discrepancy.

Discussion:

1. This seems overly long and a bit meandering. Reference to other systems and context are all well and good but the reader may give up before reaching the take home at the end.

Response: The discussion has been substantially revised based on the collective comments.
